# Soluble Epoxide Hydrolase Inhibition Improves Alzheimer’s Disease Hallmarks: Correlation with Peripheral Inflammation and Gut Microbiota Modulation

**DOI:** 10.14336/AD.2025.0201

**Published:** 2025-03-23

**Authors:** Júlia Jarne-Ferrer, Christian Griñán-Ferré, Beatrice Jora, Sandra Codony, Lluïsa Miró, Cristina Rosell-Cardona, David Miñana-Galbis, Anna Pérez-Bosque, Santiago Vazquez, Mercè Pallàs

**Affiliations:** ^1^Department of Pharmacology, Toxicology and Therapeutic Chemistry, Universitat de Barcelona, 08028 Barcelona, Spain.; ^2^Institute of Neurosciences of the Universitat de Barcelona, Passeig de la Vall d’Hebron 171, 08035 Barcelona, Spain.; ^3^Centro de Investigación Biomédica en Red Enfermedades Neurodegenerativas (CiberNed) - National Institute of Health Carlos III, 28029 Madrid, Spain.; ^4^Laboratory of Medicinal Chemistry (CSIC Associated Unit), Faculty of Pharmacy and Food Sciences, and Institute of Biomedicine (IBUB), Universitat de Barcelona, E-08028 Barcelona, Spain.; ^5^Departament de Bioquímica i Fisiologia, Facultat de Farmàcia i Ciències de l'Alimentació and Institut de Nutrició i Seguretat Alimentària, Universitat de Barcelona, 08028 Barcelona, Spain.; ^6^Secció de Microbiologia, Departament de Biologia, Sanitat i Medi Ambient, Facultat de Farmàcia i Ciències de l’Alimentació, Universitat de Barcelona, 08028 Barcelona, Spain.

**Keywords:** cognitive impairment, aging, gut-microbiota axis, neuroinflammation, soluble epoxide hydrolase

## Abstract

Targeting brain inflammation has been proposed as a promising therapeutic strategy to cope with neurodegenerative diseases. Interestingly, accumulating data suggest that the gut microbiota partially exerts its neurodegenerative effects by exacerbating neuroinflammation through increased pathogenic or unhealthy genera that releases different types of cytokines in the periphery. Recently, soluble epoxide hydrolase enzyme (sEH) emerged as a new pharmacological approach for treating Alzheimer’s Disease. Treatment with a sEH inhibitor (UB-BJ-02) modified the gut microbiota in the 5xFAD mouse model, increasing health-promoting genera such as *Lactobacillus* and *Limosilactobacillus*. By contrast, pro-inflammatory genera (e.g., *Bacteroides*) were decreased. UB-BJ-02 treatment enhanced the production of anti-inflammatory peripheral mediators in the colon and spleen, such as *Il-10*. 5xFAD mice treated with UB-BJ-02 showed improved short- and long-term memory and spatial memory compared to 5xFAD control. Furthermore, we found a reduction in neuroinflammatory markers evaluated by immunohistochemical assays, such as GFAP and IBA-1, and gene expression, such as *Il-1β, Tnf-a, Il-6*, and *Trem2*, in the brain of 5xFAD-treated mice and a significant decrease in the number of Aβ plaques. T Treatment decreased DRP1 protein levels while increasing OPA1 levels, resulting in improved mitochondrial function corroborated by the elevation of Pgc1-α. Interestingly, a correlation between UB-BJ-02 brain effects and microbiota changes were demonstrated. To validate this correlation, we fed *CL4176* AD transgenic strain, with *Limosilactobacillus reuteri* and *Bacteroides rodentium.* Consequently, we observed that changes in feeding modified the number of Aβ plaques and neuroinflammatory markers in *C. elegans*. Therefore, the present study suggested that sEH inhibition with UB-BJ-02 promoted neuroprotective effects, modulating gut microbiota and modifying peripheral and brain pro-inflammatory markers.

## INTRODUCTION

Alzheimer’s disease (AD) is the main form of dementia, which increases exponentially with age, being the most prevalent neurodegenerative disease worldwide [1]. However, until now, there have been no effective therapeutic approaches to stop the disease, only to slow it down [2]. Of note, the root cause of its pathogenesis remains unclear, and several hypotheses about it have emerged in recent years, such as the well-known amyloid hypothesis or the neuroinflammation hypothesis [3-6]. Considering this, the amyloid hypothesis defends that the extreme amyloid beta (Aβ) peptide accumulation in the brain causes AD [7]. Besides, it postulates that Aβ drives the abnormal phosphorylation of microtubule-associated Tau protein, creating neurofibrillary tangles (NFTs). Therefore, both pathological features lead to impaired neural plasticity, neuronal dysfunction, neuro-inflammation, and even neuronal death [3, 7].

Moreover, recently, Aβ has been described as an immunopeptide, concretely a cytokine, because it presents structural and functional properties that characterize these molecules, such as low molecular weight, aggregation tendency, regulation of the innate and adaptative immune responses or regulation of intercellular communications and cell signaling processes, among others [8]. This allows us to unify both previously mentioned AD hypotheses, the amyloid and neuroinflammation hypotheses, into a single process. Then, considering Aβ as a part of the neuroinflammatory process, targeting brain inflammation could be a promising and successful therapeutic target to cope with AD or other neuro-degenerative diseases.

Recently, gut dysbiosis has been identified as a potential hallmark of aging [9]. The gut microbiota, consisting of bacteria, archaea, fungi, and viruses in the gastrointestinal tract, is vital for health, influencing immune function and the gut-brain axis [10]. It supports the intestinal barrier and immune response in a balanced state. Importantly, *Bacillota (formerly Firmicutes)* and *Bacteroidota (formerly Bacteroidetes)* are the dominant gut microbial phyla, representing 90% of gut microbiota [11]. However, aging alters its composition, leading to dysbiosis characterized by increased pathogenic bacteria and a pro-inflammatory profile. This imbalance has been linked to numerous diseases, including inflammatory bowel disease and neurodegenerative diseases such as AD [12, 13]. Differences in the gut microbiota composition, characterized by an increased presence of pathogenic bacteria, have been correlated with disruptions in the integrity of the intestinal barrier [14]. Such disruptions facilitate the translocation of microbiota and their metabolic by-products across the intestinal mucosa, subsequently activating toll-like receptors (TLRs) and initiating immune responses. This inflammatory response might induce and promote neuroinflammatory responses contributing to neurodegeneration via the gut-brain axis [15].

In this way, an important enzyme in the neuroinflammation process is the soluble epoxide hydrolase enzyme (sEH), which converts the lipid mediators called epoxyeicosatrienoics acids (EETs) into their corresponding proinflammatory diols, called dihydroxyeicosatrienoic acids (DHETs). In fact, it is well-known that sEH levels are higher in the AD brains, suggesting that it could be a key enzyme in the pathogenesis of the disease, contributing to the early onset and subsequent progression of neuroinflammation. Previously, we have reported that the inhibition of sEH activity stabilizes EETs, characterized by their potent endogenous anti-inflammatory properties, among others [16]. In the brain, sEH inhibition promotes brain tissue protection, and it has been proven effective in reducing neuroinflammation, endoplasmic reticulum stress (ERS), amyloid accumulation, and p-Tau pathology, preventing memory impairment in AD mice model [17] Thus, targeting sEH could represent a therapeutic approach to AD treatment. To study further the beneficial effects of sEH inhibition, we used the 5xFAD, a mice model of familial AD that develops early and aggressive hallmarks of amyloid burden and cognitive decline, among other molecular features [18, 19]. Remarkably, recent studies revealed changes in fecal microbiota composition along with age [20], demonstrating the participation of gut microbiome in neurodegeneration presented by this animal model. Hence, we also used the *C. elegans* model, specifically the CL4176 transgenic AD strain (smg-1(cc546ts) I; dvIs27 [myo-3/Aβ minigene + rol-6(su1006)), to study the impact of the microbiome in the central nervous system (CNS). Concretely, the CL4176 strain expresses human Aβ1-42 in its muscle cells and develops a progressive paralysis phenotype [21]. Thus, this study assessed the uncovering new pathways implicated in neuroprotection by UB-BJ-02 (1-(9-Fluoro-5,6,8,9,10,11-hexahydro-7H-5,9:7,11-dimethanobenzo [9]annulen-7-yl)-3-(1-(tetrahydro-2H-pyran-4-carbonyl) piperidin-4-yl)urea) [22], a novel and selective sEH inhibitor, which is an optimization of the previous compound, called UB-SCG-51 [17, 23]. Concretely, we focused on examining the effects of sEH inhibition by UB-BJ-02 on gut microbiota, its role in modulating peripheral inflammation, and its impact on neuroinflammation, amyloid pathology, and mitochondrial dysfunction in the CNS.

## MATERIALS AND METHODS

### Animals

5-month-old male and female Wild-Type (WT Control, n=12) and 5xFAD mice (n=25) were used. 5xFAD mice were divided into 5xFAD Control (n=12) and 5xFAD treated with UB-BJ-02 at 5mg/Kg dose (5xFAD UB-BJ-02) (n=13). WT and 5xFAD control groups received vehicle ((2-hydroxypropyl)-β-cyclodextrin 1.8%) through drinking water during treatment. In the treated group, UB-BJ-02 was administered also through drinking water. The animals had free access to food and water and were kept under standard temperature conditions (22 ± 2ºC) and 12-h/12-h light/dark cycles (300 lux/0 lux).

**Figure 1.**
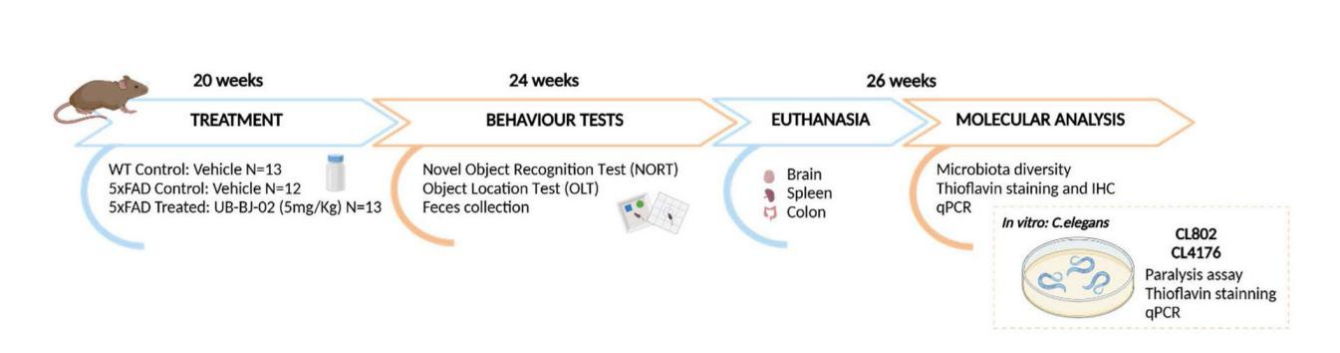
Experimental design scheme.

After 4 weeks of treatment, behavioral and cognitive tests were performed. During this period and up to the euthanasia, mice also received the vehicle or drug. All studies and procedures for mouse behavior tests, brain dissection, and extractions followed the ARRIVE and the standard ethical guidelines (European Communities Council Directive 2010/63/EU and Guidelines for the Care and Use of Mammals in Neuroscience and Behavioral Research, National Research Council 2003). They were approved by the Institutional Animal Care and Generalitat de Catalunya (#10291, 1/28/2018). All efforts were made to minimize the number of mice used and their suffering. A schematic representation of the experimental design is shown in Figure 1.

*C. elegans* were cultured according to standard procedures. CL802 strain and the transgenic AD strain CL4176 were used for this study. They were obtained from the Caenorhabditis Genetics Center (CGC), University of Minnesota, Minneapolis, MN, USA. Nematodes were maintained at 16ºC in a temperature-controlled incubator on a solid nematode growth medium (NGM) seeded with *Escherichia coli* (*E. coli*) OP50. To collect age-synchronized populations of eggs, adults were treated with an alkaline hypochlorite solution (0.5 M NaOH, ~2.6% NaCl) for 5-7 minutes. Fertilized eggs were re-suspended in S-medium for 12 hours, and L1 larvae were incubated to hatch overnight without food.

### Bacteria preparation and C. elegans treatment

#### Bacterial strains

In addition to *E. coli* OP50, *Limosilactobacillus reuteri* (*L*. *reuteri*) DSM 20016^T^ and *Bacteroides rodentium* (*B. rodentium*) DSM 26882^T^, obtained from the Leibniz Institute DSMZ-German Collection of Microorganisms and Cell Cultures GmbH, were used as a food source for *C. elegans*. *L. reuteri* and *B. rodentium* were grown on MRS agar and Columbia agar with sheep blood (Thermo Scientific, Waltham, MA, USA) under anaerobic conditions at 37ºC. To obtain concentrated bacterial cultures, *E. coli*, *L. reuteri*, and *B. rodentium* were grown overnight in Luria-Bertani (LB) broth, MRS broth, and Brucella broth (BD Diagnostics, Franklin Lakes, NJ, USA) supplemented with 10% fetal bovine serum (FBS; Invitrogen, Waltham, MA, USA), respectively. After bacterial culture centrifugation, pellets were inactivated by freeze-thaw cycles and resuspended in S-medium complete to a final optical density of 0.9-0.8 at 595 nm measured in the microplate reader. Then, synchronized nematodes were fed for 4 days with inactivated *E. coli*, *L. intestinalis*, and *B. rodentium* on a solid growth medium until they reached adulthood.

### Behavioral and cognitive tests

#### Novel Object Recognition Test (NORT)

NORT is a well-established task to study short- and long-term memory based on the mice's tendency to explore a new object more than a familiar one. The test was performed in a 90° two-arm (25 × 20 × 5 cm) black maze with removable walls for easy cleaning. First, mice were individually habituated to the apparatus for 10 minutes for 3 days. On day 4, two identical objects were located at the end of each maze arm, and the mice were allowed to explore them for 10 minutes (Familiarization trial) freely. After 2 hours and 24 hours, mice were subjected to a 10-minutes retention trial to evaluate the short-term and the long-term memory, in which one of the two old objects was replaced by a novel one. Trials were recorded, and the total exploration time was measured by analyzing the time that the mice spent exploring the new object (TN) and the time exploring the old one (TO). Exploration was defined as sniffing or touching objects with the nose and/or forepaws. Then, the discrimination index (DI) was calculated as (TN - TO)/(TN + TO). To avoid object preference biases, objects were alternated, and 70% EtOH was used to clean the arms and objects after each trial to eliminate olfactory cues.

#### Object Location Test (OLT)

The OLT is a well-established task to study the mice's spatial memory based on the spontaneous tendency of rodents to spend more time investigating an object that has been moved at a new place. The test was performed in a wooden box (50 × 50 × 25 cm), in which three walls were white except one that was black. On day 1, the box was empty, and the animals were individually habituated to the arena for 10 minutes. On day 2, two 10 cm-high-identical objects were placed in front of the black wall, equidistant from each other and the wall. Then, the animals were placed into the arena and allowed to explore objects and surroundings for 10 minutes freely. After, the mice were returned to their cages, and the OLT apparatus was cleaned with 70% ethanol. On day 3, after 24 hours, one object was moved in front of the white wall, and again, the mice were placed into the arena and allowed to explore it for 10 minutes. Trials were recorded, and the total exploration time was evaluated, considering the amount of time (seconds) spent sniffing the object in the TN and the object in TO. A DI was calculated to determine cognitive performance, which is defined as (TN-TO)/(TN+TO).

#### Paralysis assay

Synchronized worms fed with the corresponding bacteria were maintained at 16ºC for 2 days. Next, the temperature was upshifted to 25ºC. Paralysis of the worms was scored 20 hours after the initiation of upshift, and a continued scoring in two-hour increments was performed. Worms that have recently initiated paralysis cannot translate across the plate but can move their heads, clearing bacteria around their anterior and leaving a "halo" of cleared bacteria. These worms with halos were categorized as paralyzed. Some worms will not have halos but will also not show spontaneous movement; these were tested by prodding with the worm picker. If a prodded worm cannot undergo a complete body wave propagation upon prodding, it was scored as paralyzed. The percentage of worms not paralyzed every 2 hours was calculated.

### Biochemical experiments

#### Samples obtention

Feces were collected in clean conditions after behavior studies. Samples were immediately frozen in liquid N_2_ and maintained at -80ºC until use. At the end of the collection, mice were anesthetized by an i.p. injection of ketamine/xylazine (100/10 mg/kg) dissolved in 0.9% saline. Mice were euthanized by cervical dislocation; blood was collected directly from the heart and the brain was quickly removed from the skull. Afterward, the hippocampus and cortex were dissected, frozen in powdered dry ice, and maintained at -80ºC for further biochemical experiments. Spleen and colon mucosa were dissected and frozen at -80ºC for future use.

#### Protein levels determination by Western blotting (WB)

For WB, tissue samples were homogenized in a lysis buffer containing phosphatase and protease inhibitors (Cocktail II, Sigma-Aldrich, St. Louis, MO, USA), and protein concentration was determined by Bradford’s method.

Aliquots of 15 μg of protein samples from 18 mice of both strains (n=6 per group) were separated by Sodium dodecyl sulfate-polyacrylamide gel electrophoresis (SDS-PAGE) (8-14%) and transferred onto polyvinylidene difluoride membranes (PVDF, Millipore). Afterward, membranes were blocked in 5% Bovine Serum Albumin (BSA) in 0.1% Tris-buffered saline with Tween 20 (TBS-T) for 1 hour at room temperature, followed by the overnight incubation at 4ºC with the primary antibodies presented Supplementary Table 1.

The next day, membranes were washed 3 times for 5 minutes with Tris-buffered saline with Tween® 20 Detergent (TBS-T) and incubated with secondary antibodies (mouse or rabbit) for 1 hour at room temperature. Chemiluminescence-based detection was used to view the immunoreactive proteins, following the manufacturer’s protocol (ECL Kit, Millipore). Then, digital images were acquired using an Amersham Imager 680, and semiquantitative analyses were performed using ImageLab Software (BioRad, Hercules, CA, USA). Finally, results were expressed in Arbitrary Units (AU), considering the WT control mice group as 100%. Protein loading was routinely monitored by immunodetection of GAPDH. In the same way, for the phosphorylated protein ratio, normalization against GADPH was done before the ratio value calculation.

#### RNA extraction and gene expression determination

Samples of brain, spleen, colon and adult worms were used to obtain RNA. Total RNA isolation was carried out using a Trizol reagent following the manufacturer’s instructions. The samples' yield, purity, and quality of the RNA content were measured at 260 nm and determined spectrophotometrically by an A260/280 ratio using a NanoDrop™ ND-1000 (Thermo Fisher Scientific, Waltham, MA, USA). Also, to determine the RNA integrity number, samples were examined in an Agilent 2100B Bioanalyzer (Agilent Technologies, Santa Clara, CA, USA). RNAs with 260/280 ratios and RIN higher than 1.9 and 7.5, respectively, were selected.

Afterward, a reverse transcription-polymerase chain reaction (RT-PCR) was performed. We use a High-Capacity cDNA Reverse Transcription kit (Applied Biosystems, Foster City, CA, USA) to reverse-transcribe 2 μg of messenger RNA (mRNA). Then, a Real-time PCR (qPCR) was performed on the Step One Plus Detection System (Applied Biosystems, Waltham, MA, USA) using the SYBR Green PCR Master Mix (Applied Biosystems). Concretely, each reaction mixture contained 6.75 μL of cDNA (2 μg concentration), 0.75 μL of each primer (100 nM concentration), and 6.75 μL of SYBR Green PCR Master Mix (2X). The comparative cycle threshold (Ct) method was used to analyze the data, where the β-actin transcript level in each sample was used to normalize differences in sample loading and preparation. All primers used in this work are listed in Supplementary Table 2. Each sample was analyzed in duplicate, and the results represented the n-fold difference in transcript levels compared to control group.

#### Aβ plaques histology

The brains were fixed in 4% PFA overnight at 4 °C and were changed to PFA + 15% sucrose the following day. Finally, brains were frozen in isopentane and stored at - 80 °C. The frozen brains were embedded into an OCT Cryostat Embedding Compound (Tissue-Tek, Torrance, CA, USA) and then cut into 30-μm-thick sections at -20ºC using a cryostat (Leica Microsystems, Germany). For the Thioflavin-S (Th-S) staining procedure, three brain sections per animal were first rehydrated with PBS 1x at room temperature for 5 minutes. Afterward, brain sections were washed sequentially with 50%, 70%, and 80% ethanol. Immediately, slices were incubated with 0.3% Th-S (Sigma-Aldrich) solution for 10 minutes at room temperature in the dark. Subsequently, these samples were submitted to 1-minute washes of 80%, 70%, and 50% ethanol, and finally, one more wash of 1 mL of PBS 1x for 5 minutes. Then, slides were mounted with Fluoromount-GTM (EMS, Hatfield, NJ, USA) and allowed to dry overnight in the dark. Image acquisition was performed with a fluorescence laser microscope (Olympus BX51; Germany) using a 20x objective. For plaque quantification, we used ImageJ software, and similar and comparable histological areas were selected, focusing on the adjacent positioning of the whole cortical area and the hippocampus. Each image was converted to 8-bit greyscale, thresholded to a linear scale, and the number of particles covered by Th-S was calculated and then averaged from the three different sections of each animal.

Adult worms were fixed in 4% Parafor-maldehyde/PBS (pH 7.5) for 24 hours at 4ºC. The next day, worms were permeabilized in 5% fresh β-mercaptoethanol, 1% Triton X-100, and 125 mm Tris (pH 7.5) at 37ºC for another 24 hours. Then, nematodes were stained with 0.125% Th-S (Sigma) in 50% ethanol for 2 minutes, destained in 50% ethanol for 2 minutes, and washed 3 times with PBS. Approximately 10 µL of Fluoromount G was used to prepare the glass slide for microscopy (Electron Microscopy Sciences). Fluorescence images were acquired using a 20Å~ objective of a fluorescence microscope. Aβ in the head region of worms was quantified unthinkingly by counting the number of favourable Th-S spots using ImageJ.

#### Immunofluorescence assay

Brains were fixed, frozen, cut and preserved as previously mentioned in mice Aβ plaques histology. For immunohistochemical assays, free-floating brain slices were washed three times for 5 min in PBS (0.1M) and blocked and permeabilized in PBS, BSA 1% and 0,3% Triton X-100 solution for 20 min. After two washes of 5 min with PBS, we incubated the primary antibodies GFAP (Abcam/ab279289) and IBA-1 (Genetex/6TX100042) over-night at 4ºC at a dilution of 1:400. The following day, after two washes with PBS, the secondary antibodies (Alexa Fluor 488 Goat anti-Mouse and Alexa Fluor 555 Donkey anti-Rabbit) were incubated at room temperature for 1 h in the dark at a dilution of 1:400 for GFAP and 1:200 for IBA-1. Then, brain sections were co-incubated also at room temperature with 1 mg/mL Hoechst (Sigma) staining solution for 5 min in the dark and washed three times for 5 min in PBS. Finally, the slices were mounted with Fluoromount G (EMS, USA). Immunohistochemistry images were performed with a fluorescence laser microscope (Olympus BX51, Germany). A minimum of four sections from three different individuals per group were analyzed using ImageJ/Fiji software (National Institutes of Health, online). For GFAP and IBA1 image acquisition, a consistent exposure setting was applied across all samples and experiments. Fluorescence intensity of GFAP- and IBA1-positive cells was measured in distinct regions of the hippocampus (dentate gyrus, *cornu ammonis* 1 (CA1) and CA3), with the quantification averaged across the different sections from each subject.

#### Quantification of plasma cytokines

The concentrations of the cytokines interleukin-1β (IL-1β) and tumor necrosis factor-alpha (TNF-α) in plasma samples were determined using the Bio-Plex Cytokine Assay™ (Bio-Rad, Hercules, CA, USA), according to the manufacturer’s instructions.

#### DNA extraction and 16S rRNA gene sequencing

DNA was extracted according to Moreto et al [24]. Briefly, microbial cells from fecal samples (70 mg/mouse) were mechanically disrupted by bead-beating (FastPrep®-24) with zirconia/silica beads in the presence of guanidine thiocyanate and N-lauroyl sarcosine. After washing and purification steps, nucleic acids were precipitated, treated with RNase, and finally resuspended in water for quantification via spectrophotometry (NanoDrop ND-100). DNA samples were analyzed on the Illumina 16S rRNA sequencing platform. The V3 and V4 hypervariable regions of the bacterial 16S rRNA gene were amplified by PCR, using specific primers: forward 5′-TCGTCGGCAGCGTCAGATGTGTATAAGAGAC AGCCTACGGGNGGCWGCAG-3′ and reverse 5′ -GT CTCGTGGGCTCGGAGATGTGTATAAGAGACAGGACTACHVGGGTATCTAATCC-3′. High-through sequencing was done using the Illumina MiSeq platform (Illumina, San Diego, CA, USA) at the Genomics and Bioinformatics Service, Universitat Autònoma de Barcelona (Bellaterra, Spain).

### Microbiota analysis

Microbiome bioinformatics was performed with QIIME2 2023.7 [25]. Raw sequence data were demultiplexed and quality filtered using the q2-demux plugin, followed by denoising with DADA2 [26]. All amplicon sequence variants (ASVs) were aligned with mafft [27] and used to construct a phylogeny with FastTree2 [28]. α -diversity metrics (Shannon index and Faith’s Phylogenetic Diversity) and β -diversity metrics like Bray-Curtis dissimilarity was estimated using q2-diversity after rarefied samples [29, 30]. Taxonomy was assigned to ASVs using the q2-feature-classifier [31] classify-sklearn naive Bayes taxonomy classifier against the Greengenes2 reference sequences [32].

### Statistical analysis

Data are expressed as mean ± standard error of the mean (SEM). Statistical analysis was performed using GraphPad Prism version 9 (GraphPad Software, Inc., La Jolla, CA, USA). First, Grubb’s test was performed to detect outliers, which were removed from the analysis, in addition to Levene’s test to check the homogeneity of variance and the Shapiro-Wilk test to verify data normality across all groups. To compare groups, one-way ANOVA followed by the Fisher or Tukey’s posthoc test were used for normally distributed data. In contrast, the Kruskal-Wallis test was used for non-normally distributed data. Statistical significance was considered when P values were <0.05.

Microbiota analysis only included taxa with a percentage of reads higher than 0.001%. Permutation Multifactorial Analysis of Variance (PERMANOVA) was performed using the Bray-Curtis dissimilarity matrix by the “adonis” function in R. Principal coordinate analysis (PCoA) ordination based on Bray-Curtis dissimilarity was used to visualize the dispersion of microbial community among groups. The linear discriminant analysis effect size (LEfSe) method with logarithmic discriminant analysis (LDA) scores was used to identify taxa differing relative abundance among groups. For LEfSe analysis, the Kruskal-Wallis test, an all-against-all strategy, was applied with a P < 0.05 and a logarithmic LDA score threshold of 2.0. Using the Vegan package, both analyses were performed in RStudio (2023.09.1 Build 494^©^ 2009-2023 Posit Software, PBC). Correlation analysis was performed to assess the relationships between gut microbiome composition, immune response markers, metabolite concentrations, and measures of cognitive function. Spearman's rank correlation test was used, and correlation coefficients were calculated for each pair of variables. A two-tailed p-value of less than 0.05 was considered statistically significant. Subsequently, the correlation coefficients were visualized using a heat map. Both the correlation and its plotting were performed using the R package corrplot. The significant Spearman's rank correlation coefficients were utilized to delineate the connections between various entities designated as nodes within the network and construct the network diagram. These nodes corresponded to distinct bacterial genera identified in the gut microbiota, quantified immune response markers, measured metabolite concentrations, and evaluated metrics of cognitive function. Cytoscape version 3.9.1, a specialized software tool for complex network analysis and visualization [33], facilitated the visualization of the network.

**Figure 2.**
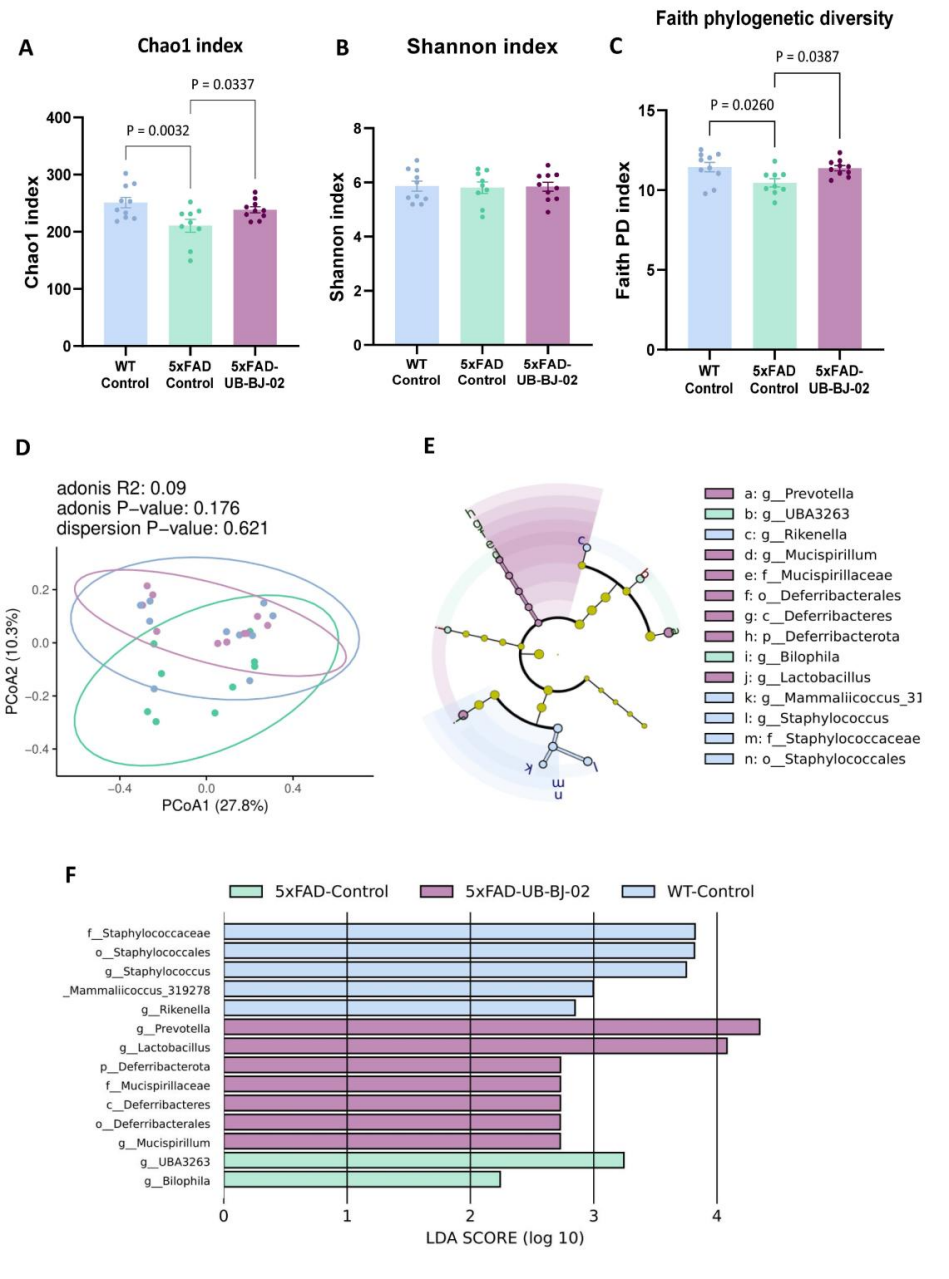
**Microbiota richness and diversity in WT Control, 5xFAD Control, and 5xFAD UB-BJ-02 groups**. (**A**) Microbiota richness (Chao1 index), (B-C) α-diversity; Shannon index, panel B; Faith’s Phylogenetic Diversity, panel C). Groups were compared using the One-Way ANOVA test and Fisher posthoc test for α-diversity comparisons. (**D**) β-diversity (Principal coordinates Analysis based on Bray-Curtis distance). Ellipses show 95% confidence intervals. PERMANOVA was used to assess the statistical significance of β-diversity comparisons. (**E**) A cladogram using the LEfSe method indicates the phylogenetic distribution of fecal microbiota. Each successive circle represents a phylogenetic level. (**F**) A histogram of linear discriminant analysis (LDA) scores reveals the most differentially abundant taxa among different groups. Number of animals (8-11 mice/group).

## RESULTS

### sEH inhibition influences the microbial composition

#### Microbiota richness and diversity

In assessing microbiome richness, we employed the Chao1 index, a non-parametric model, to provide a conservative estimation of total ASV richness for each subject in our murine model. Our results indicate a diminished microbiome richness in 5xFAD mice, as evidenced by a significantly lower Chao1 index (P = 0.0032, Fig. 2A). Conversely, administration of UB-BJ-02 in 5xFAD mice showed a notable enhancement of microbiome richness (P = 0.0337).

To evaluate α-diversity, we utilized two distinct metrics: the Shannon Index and Faith’s Phylogenetic Diversity (PD), with the latter integrating phylogenetic relationships into the diversity assessment. While our analysis revealed no significant alteration in the Shannon Index (Fig. 2B), Faith’s PD demonstrated a substantial decrease in the 5xFAD-Control group (P = 0.026; Fig. 2C), which suggested a loss of phylogenetic diversity. Notably, UB-BJ-02 treatment effectively mitigated this loss in 5xFAD mice.

**Figure 3.**
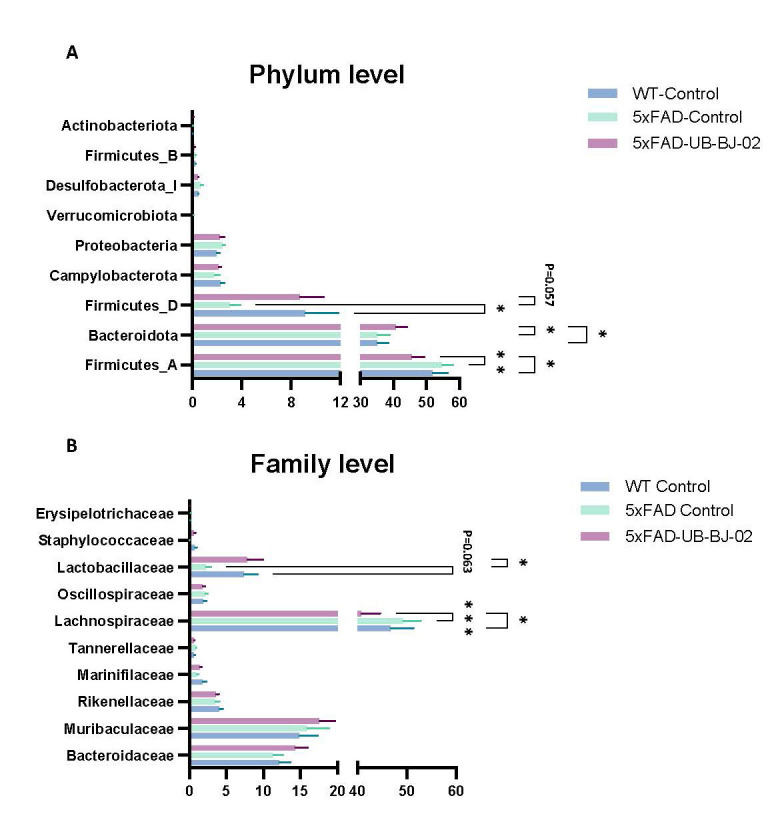
**Differential abundance of gut microbiota at (A) the phylum and (B) family level in WT Control, 5xFAD Control, and 5xFAD UB-BJ-02 groups**. Results are expressed as mean ± SEM (n = 8-11 mice/group). Groups were compared using the One-Way ANOVA test and Fisher posthoc test. Asterisks indicate statistical significance between groups, *p < 0.05, **p < 0.01, and ***p < 0.001.

**Figure 4.**
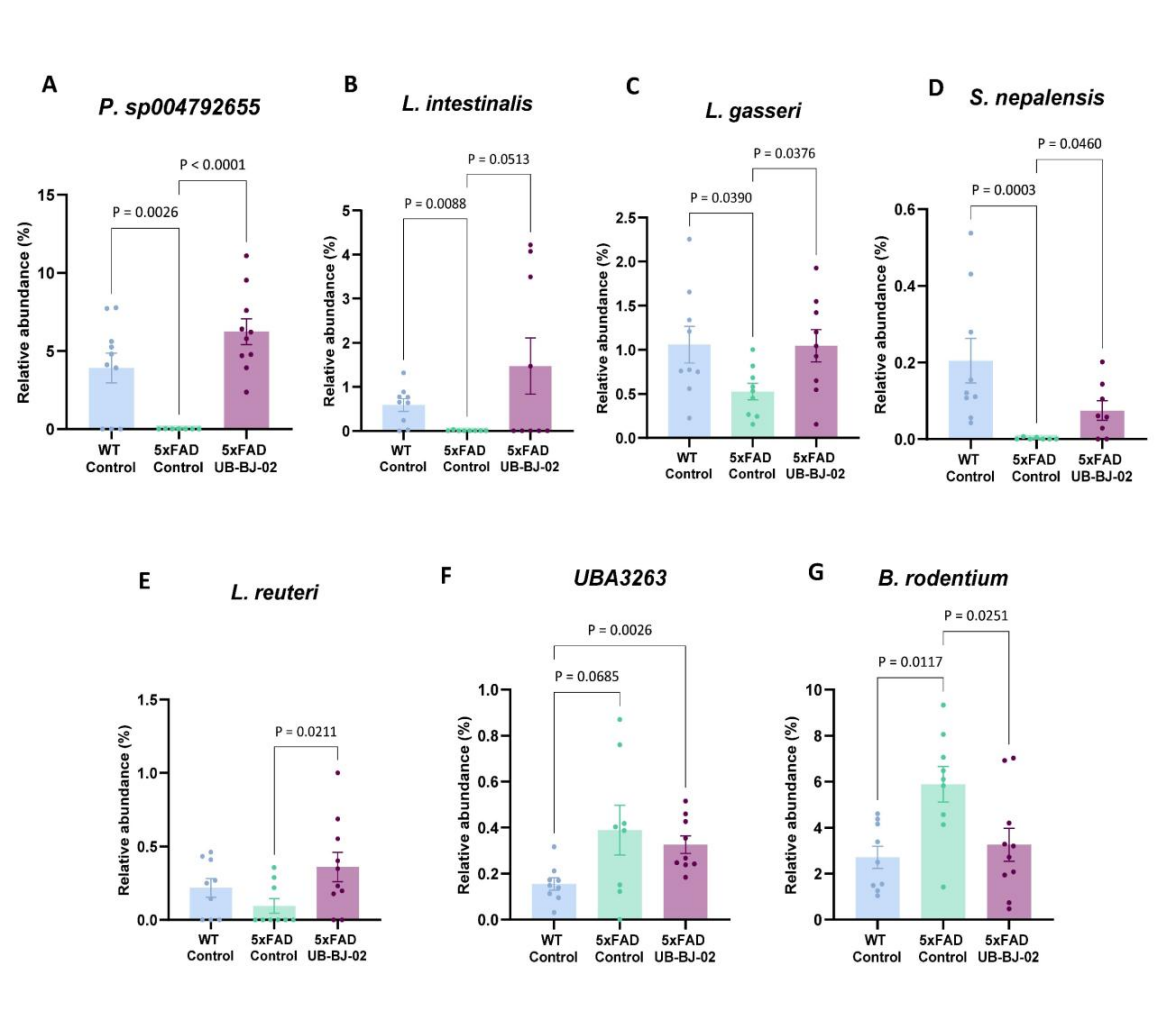
**Differential relative abundance at genus level between WT Control, 5xFAD Control, and 5xFAD UB-BJ-02 groups**. (**A**) *Prevotella sp004792655*, (B) *Lactobacillus intestinalis*, (C) *Lactobacillus gasseri*, (D) *Staphylococcus nepalensis*, (E) *Limosilactobacillus reuteri*, (F) UBA3263, and (G) *Bacteroides rodentium*. Results are expressed as mean ± SEM (n = 8-11 mice/group). Groups were compared using the One-Way ANOVA test and Fisher posthoc test.

Regarding β-diversity, the Bray-Curtis dissimilarity index was applied to compare the microbial structures across different experimental groups. This analysis yielded non-significant differences (Fig. 2D), implying a similar microbial composition among the groups. Furthermore, we explored the differential abundance of taxa using LEfSe analysis. This approach highlighted significant disparities in the abundance of specific taxa among the groups. In particular, the genera *Prevotella* and *Lactobacillus* were predominantly abundant in the 5xFAD-UB-BJ-02 group. In contrast, the genera UBA3263 and *Bilophila* were significantly enriched in the 5xFAD-Control group. These differences were substantiated by LDA scores (log10) exceeding the threshold of 2.0 (Fig. 2E, F).

#### 3.1.2 Microbiota composition

In our analysis of bacterial abundance at the phylum level, significant changes were observed predominantly in the main bacterial phyla, namely *Bacillota* (formerly *Firmicutes*) and *Bacteroidota* (formerly *Bacteroidetes*) (Fig. 3A). Specifically, while the mouse strain itself did not significantly affect the relative abundance of Firmicutes_A, treatment with UB-BJ-02 notably decreased their relative percentage in comparison to both 5xFAD-Control mice (P = 0.0012) and WT-Control mice (P = 0.0226). Conversely, the relative abundance of Bacteroidota increased in mice treated with UB-BJ-02 compared to both WT-Control and 5xFAD-Control mice, with the increase being statistically significant in the former (P = 0.0369) but not in the latter (P = 0.412). Furthermore, for Firmicutes_D, a reduction in its percentage was observed in 5xFAD mice (P = 0.0453), whereas UB-BJ-02 treatment led to an increase, nearing statistical significance (P = 0.0569).

When examining the distribution of prominent families within the fecal microbiota, significant changes were detected in the families of *Lachnospiraceae* (within the phylum Firmicutes_A) and *Lachnospiraceae* (within the phylum Firmicutes_D; Fig. 3B). Treatment with UB-BJ-02 significantly reduced the percentage of Lachnospiraceae compared to 5xFAD-Control (P = 0.0007) and WT-Control mice (P = 0.0144). In the case of *Lachnospiraceae*, there was a noticeable decrease in its relative percentage in 5xFAD mice (P = 0.0625), while UB-BJ-02 treatment led to an increase (P = 0.0361).

Our results outline distinct microbial shifts after LEfSe analysis to identify differentially abundant genera. In 5xFAD mice, there was a significant depletion in the relative abundance of *Prevotella sp004792655*, *Lactobacillus intestinalis*, *Lactobacillus gasseri*, and *Staphylococcus nepalensis* (P = 0.0026, P = 0.0088, P = 0.0390 and P = 0.0003, respectively; Fig. 4A-D). Treatment with UB-BJ-02 significantly reversed the observed depletion in these genera (P < 0.0001, P = 0.0513, P = 0.0376, and P = 0.0460, respectively), indicative of a preventive effect of the treatment on these microbial populations. Furthermore, UB-BJ-02 treatment also increased the abundance of *Limosilactobacillus reuteri* (P = 0.0211, Fig. 4E), a beneficial genus within the *Lachnospiraceae* family.

On the other hand, 5xFAD mice exhibited an elevated relative abundance of the genera *UBA3263* and *Bacteroides rodentium* (P = 0.0685 and P = 0.0117, respectively; Fig. 4F-G). Notably, UB-BJ-02 treatment significantly reduced the relative abundance of *B. rodentium* (P = 0.0251).

**Figure 5.**
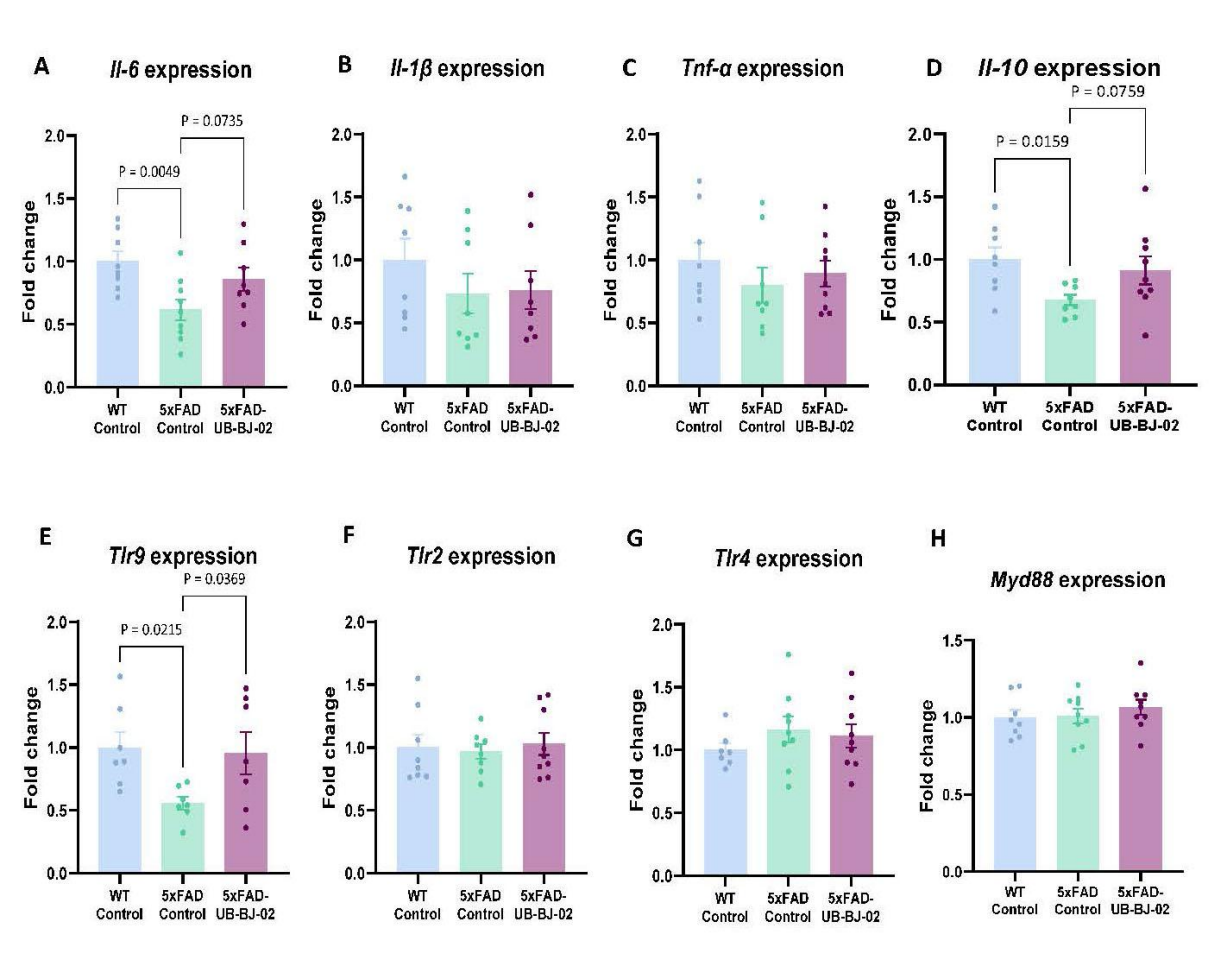
**Differential colon gene expression in WT-Control, 5xFAD-Control, and 5xFAD-UB-BJ-02 groups**. Expression of (A) *Il-1β*, (B) *Il-6*, (C) *Tnf-α*, (D) *Il-10*, (E) *Tlr2*, (F)*Tlr4*, (G) *Tlr9* and (H) *Myd88*. Results are expressed as mean ± SEM (n = 7-9 mice/group). Groups were compared by the One-Way ANOVA test and Fisher posthoc test.

### sEH inhibition by UB-BJ-02 promotes an anti-inflammatory effect in the gastrointestinal tract

In the colon mucosa of 5xFAD mice, the expression of *Il-6* was reduced (P = 0.0049, Fig. 5A). At the same time, treatment with UB-BJ-02 demonstrated a trend toward mitigating the reduction in *Il-6* expression, although this effect did not reach statistical significance (P = 0.073). The expression of other pro-inflammatory cytokines, such as *Il-1β* and *Tnf-α*, was not altered by mice strain or after UB-BJ-02 treatment (Fig. 5B, C). On the other hand, the expression of *Il-10*, an anti-inflammatory cytokine, was significantly reduced in the colon mucosa of 5xFAD mice (P = 0.0159, Fig. 5D). Notably, the UB-BJ-02 treatment showed a trend towards increasing the expression of *Il-10* (P = 0.076), suggesting a potential anti-inflammatory effect of this treatment.

Regarding *Toll-like receptor (TLR)* expression, we observed a specific downregulation of the *Tlr9* receptor in the colon mucosa of 5xFAD mice, which was statistically significant (P = 0.0215, Fig. 5E). The administration of UB-BJ-02 effectively prevented this downregulation (P = 0.0369). However, *Tlr2, Tlr4*, and *Myd88* expressions were not significantly altered in the 5xFAD mice or affected by the UB-BJ-02 treatment (Fig. 5F-H).

**Figure 6.**
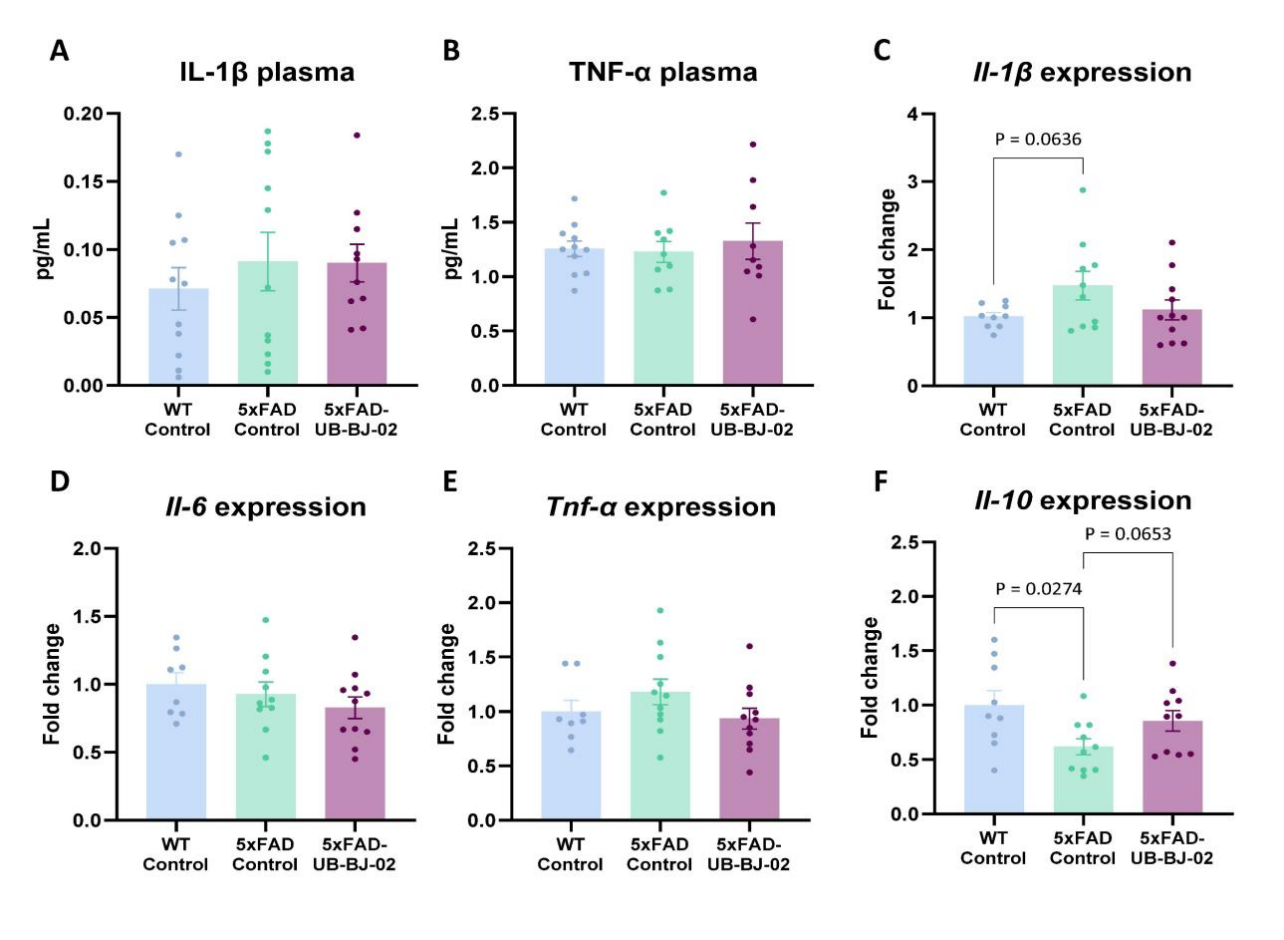
**Systemic inflammatory markers in WT-Control, 5xFAD-Control, and 5xFAD-UB-BJ-02 groups**. Plasma concentration of (A) IL-1β and (B) TNF-α. Gene expression of (C) *Il-1b*, (D) *Il-6*, (E) *Tnf-a*, and (F) *Il-10* in spleen tissue. Results are expressed as mean ± SEM (n = 8-11 mice/group). Groups were compared using the One-Way ANOVA test and Fisher posthoc test.

### Systemic action of UB-BJ-02 after sEH inhibition

Our findings revealed that this treatment did not significantly alter plasma levels of IL-1β) or TNF-α (Fig. 6A, B). In contrast, a notable change was observed in the expression of pro-inflammatory cytokines within spleen tissue. Specifically, there was an elevated expression of *Il-1β* in the spleens of 5xFAD mice, approaching statistical significance (P = 0.064, Fig. 6C). However, the expression of *Il-6* and *Tnf-α* in the spleen was not significantly affected (Fig. 6D, E). Furthermore, the anti-inflammatory cytokine *Il-10* expression was significantly reduced in the spleen of 5xFAD mice (P = 0.0274, Fig. 6F). This reduction in anti-inflammatory response is noteworthy and may affect the overall inflammatory state in 5xFAD mice. Notably, the oral administration of UB-BJ-02 appeared to counteract this effect, as evidenced by a trend toward normalized *Il-10* expression level (P = 0.065). This finding suggests a potential protective role of UB-BJ-02 in maintaining anti-inflammatory cytokine levels within the spleen of 5xFAD mice.

### sEH inhibition improves cognition and reduces β-amyloid burden in 5xFAD mice

**Figure 7.**
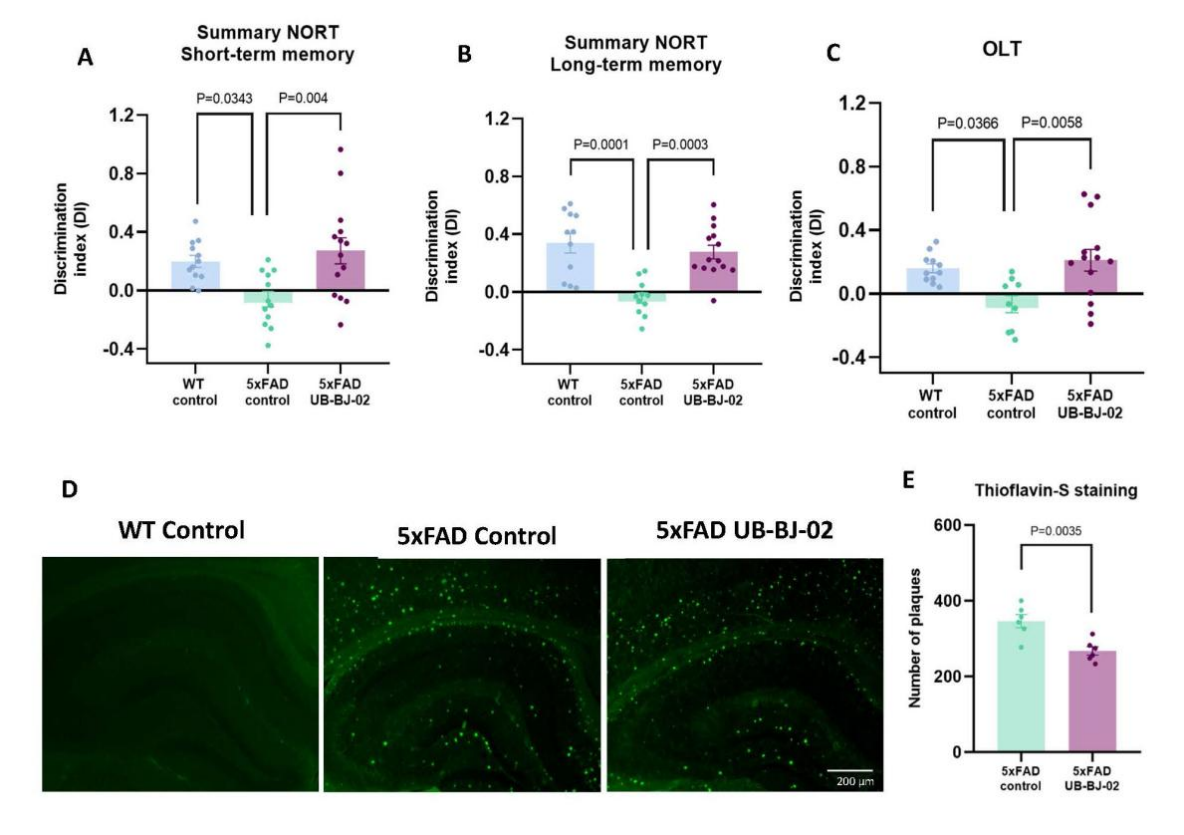
**Behavioral results and reduction of Aβ plaques**. (**A**) Short-term memory test at 2 hours and (B) long-term memory test at 24 hours in NORT. (**C**) Spatial memory test in OLT. Results are expressed as a mean SEM. Groups were compared by the One-Way ANOVA test and post-hoc Tukey’s test in NORT and OLT (n=12-13/group). (**D**) Representative images of Aβ plaques stained with Th-S in the hippocampus of WT Control, 5xFAD Control, and 5xFAD UB-BJ-02 mice. (**E**) Quantitative analysis of Th-S staining. Groups were compared by one-tailed Student’s t-test (n=6/group).

NORT was done to study the working memory after UB-BJ-02. 5xFAD treated mice showed an improvement in short- and long-term memory, presenting a significantly higher DI compared with 5xFAD control mice (P = 0.004 and P = 0.0003, Fig. 7A, B), meaning that the treated mice explored the new object more time. We also performed the OLT, which allows us to evaluate spatial memory. 5xFAD UB-BJ-02 mice presented significantly higher DI compared with the 5xFAD control (P = 0.0058, Fig. 7C). Therefore, results showed that sEH inhibition by UB-BJ-02 prevents the 5xFAD mice cognitive impairment.

Because 5xFAD mice presented Aβ aggregates, forming plaques, Th-S staining evaluates the number of plaques to study neuroprotective effects. As expected, WT brain animals did not present Aβ aggregates, whereas 5xFAD mice control exhibited an elevated number of Aβ plaques in the cortex and hippocampus. Nevertheless, 5xFAD treated with UB-BJ-02 presented a lower number of plaques in comparison with 5xFAD control; concretely, a significant reduction of 29% in Aβ plaques was found (P = 0.0035, Fig. 7D, E).

### sEH inhibition by UB-BJ-02 induces changes in neuroinflammatory markers

**Figure 8.**
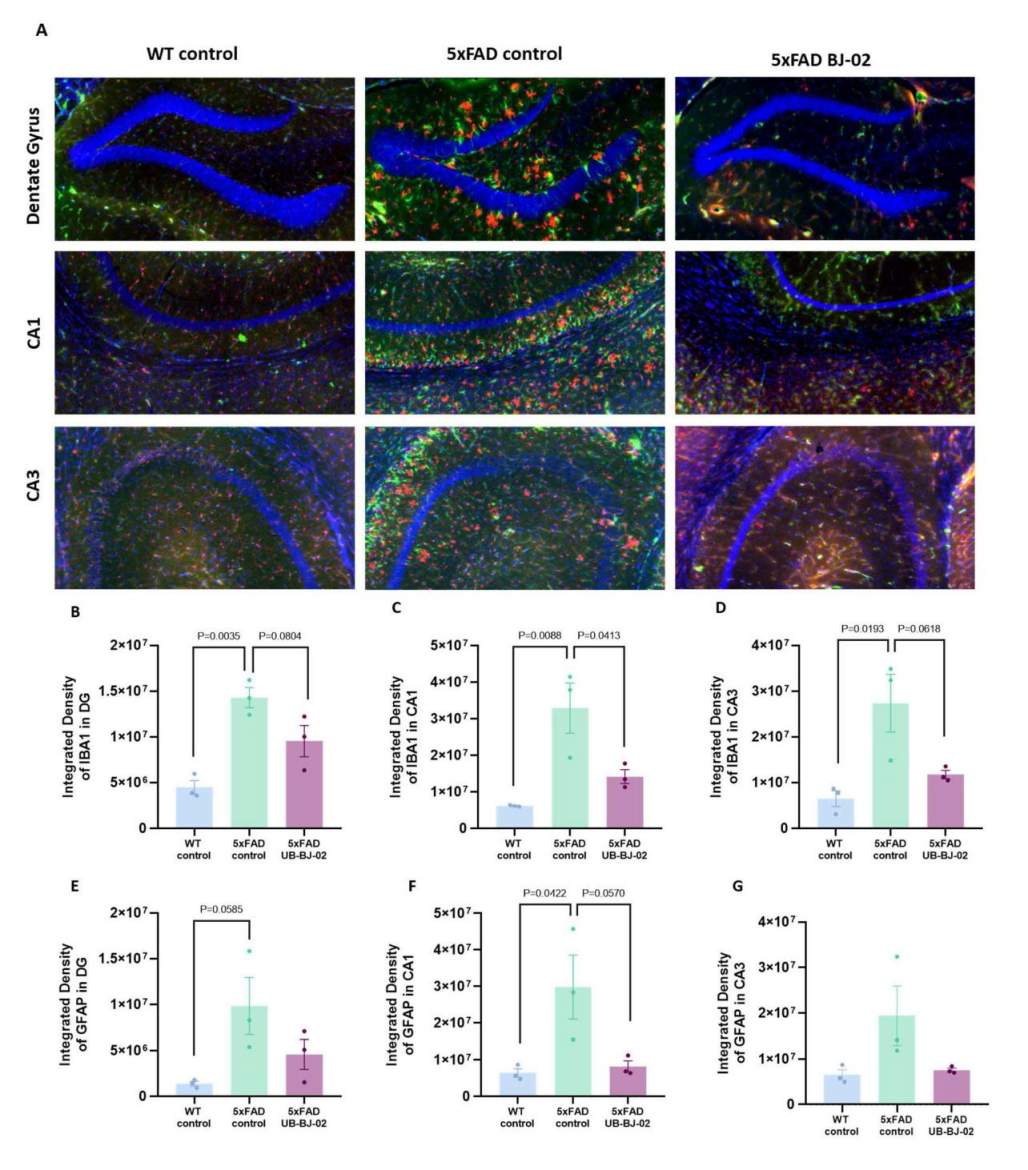
**Subtitle here**. (**A**) Representative images for IBA-1 and GFAP immunostaining and its relative expression quantification in (B, E) *dentate gyrus*, (C, F) *cornu ammonis* (CA) 1 and (D, G) CA3. Results are expressed as a mean SEM. Groups were compared using the One-Way ANOVA test and post-hoc Tukey’s test (n=3/group).

Evidence of microglial and astrocyte activation in AD brains highlights the significant role of neuro-inflammation in AD pathogenesis, contributing to a pro-inflammatory environment that exacerbates neuronal damage and accelerates disease progression [34, 35].. Then, 5xFAD reactive microglia and astroglia were evaluated by immunofluorescence analysis of ionized calcium-binding adapter molecule 1 (IBA-1) and Glial fibrillary acidic protein (GFAP) expression, respectively (Fig. 8A).

We demonstrated a higher expression of GFAP and IBA-1 in 5xFAD mice compared to WT mice in all hippocampal regions: dentate gyrus (DG), cornu ammonis 1 (CA1) and CA3 (P=0.0035, P=0.008, P=0.0193, P=0.0585, P=0.042, Fig. 8). Interestingly, UB-BJ-02 treatment attenuated microgliosis in the hippocampus of 5xFAD brains by decreasing IBA-1 immunoreactivity (P=0.0804, P=0.0618, Fig. A-D), reaching significance in CA1 (P=0.0413, Fig. 8C). In the same way, the treatment slightly reduced GFAP immunostaining in the dentate gyrus, CA3, and especially in CA1 (P=0.0570, Fig. 8A, E-G), demonstrating a reduction of hippocampal astrogliosis.

**Figure 9.**
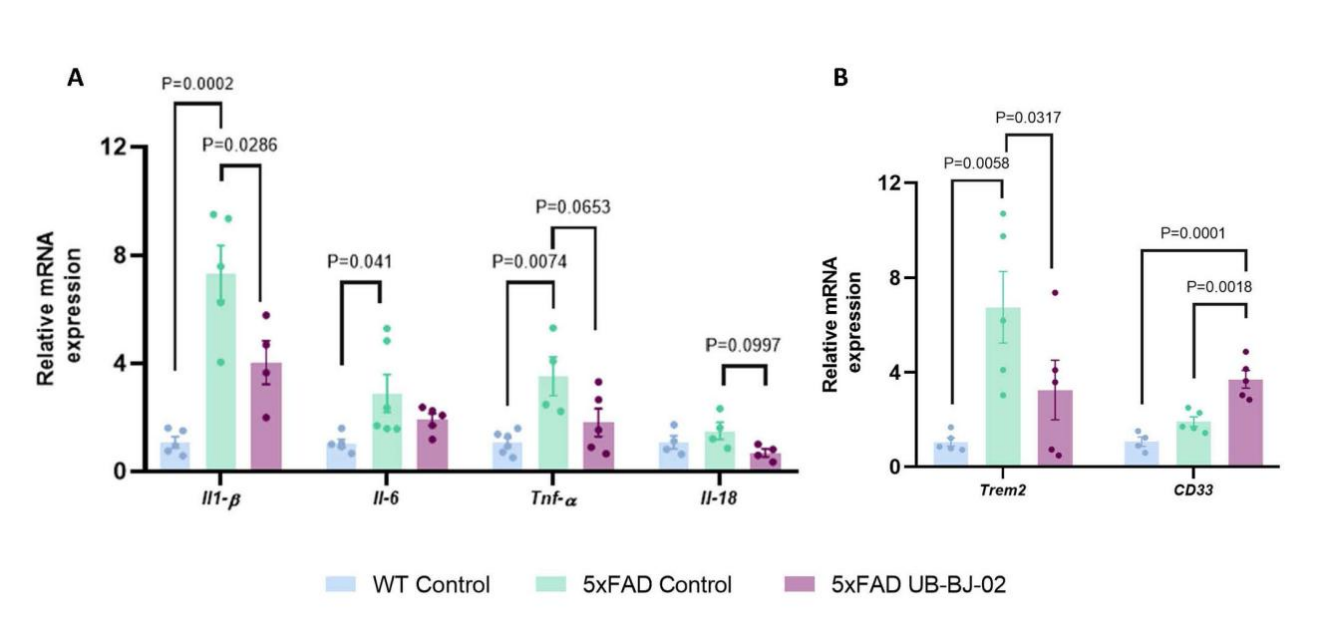
**Neuroinflammatory markers after UB-BJ-02 treatment**. Representative gene expression levels of (A) *Il-1β, Il-6, Tnf-α, and Il-18, (B) Trem2*, and *CD33*. Results are expressed as a mean SEM. Groups were compared using the One-Way ANOVA test and post-hoc Tukey’s test (n=6/group).

In this line, pro-inflammatory markers *Il-1β*, *Il-6*, *Tnf-α*, and *Il-18* gene expression were analyzed. We observed that 5xFAD control animals presented higher gene expression of those pro-inflammatory markers compared to the 5xFAD UB-BJ-02 mice (P = 0.0286, P= 0.249, P = 0.0653, P = 0.0997, respectively; Fig. 9A). Furthermore, microglial receptors *triggering receptor expressed on myeloid cells 2 (Trem2*) and *sialic acid binding Ig-like lectin 3* (*CD33*), associated with risk for AD, were also analyzed. A significative reduction of Trem2 in 5xFAD mice treated compared to 5xFAD control (P = 0.0317, Fig. 9B). In contrast, *CD33* gene expression was significantly higher in 5xFAD UB-BJ-02 compared with 5xFAD control animals (P = 0.0018, Fig. 8B). Therefore, our results highlighted the effectiveness of sEH inhibition in attenuating microgliosis and astrogliosis, modulating the neuroinflammatory process.

### sEH inhibition modulates mitochondrial dysfunction in the brain

**Figure 10.**
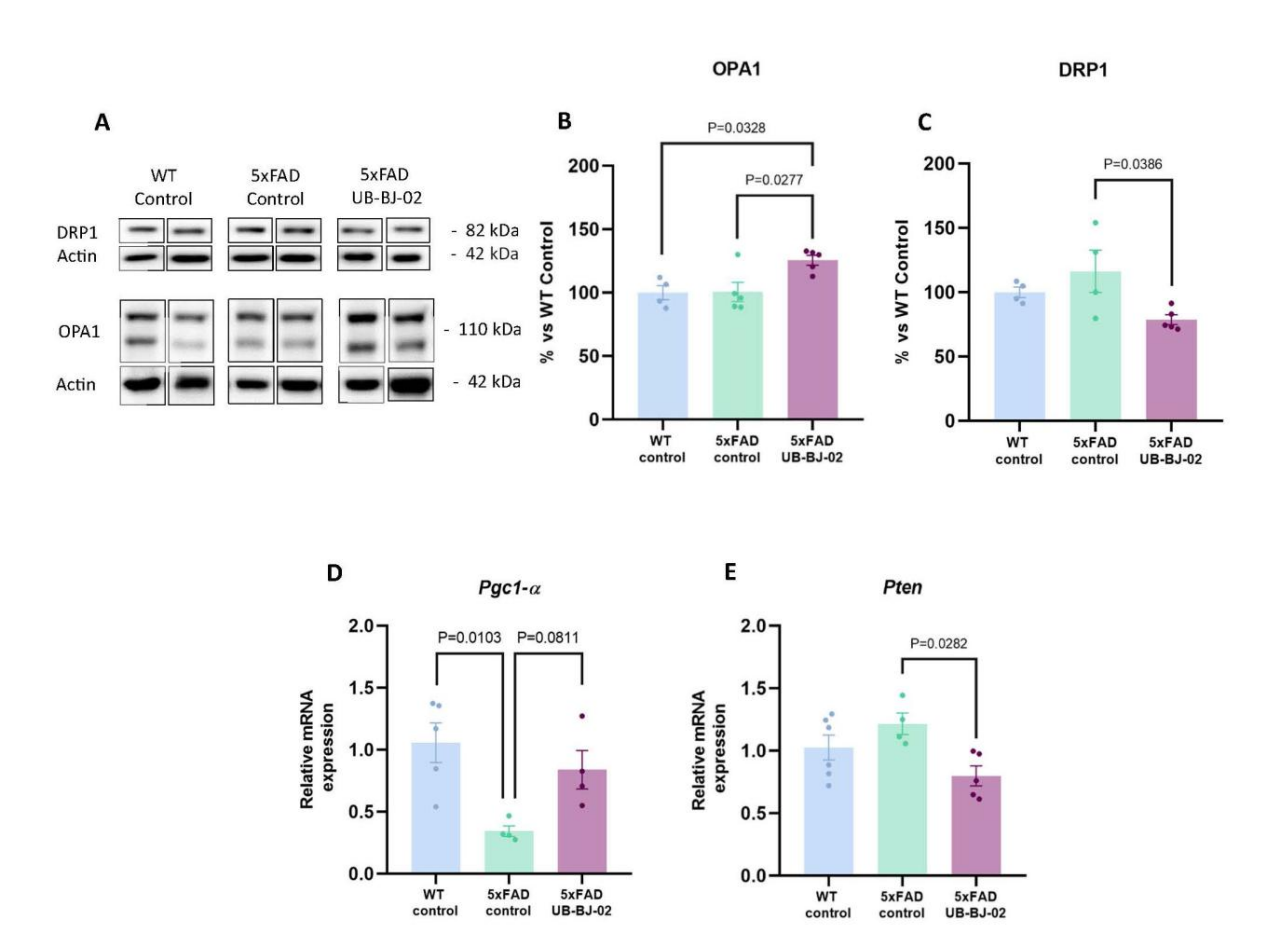
**Mitochondrial dysfunction analysis after UB-BJ-02 treatment**. (**A**) Immunoblots and representative quantification of (B) OPA1 and (C) DRP1. Representative gene expression levels of (D) *Pgc1-α* and (E) *Pten*. Results are expressed as a mean SEM. Groups were compared using the One-Way ANOVA test and post-hoc Tukey’s test (n=6/group).

Mitochondria are the principal source of intracellular energy and regulate cell survival and death. Moreover, mitochondria also produce reactive oxidative species (ROS), one of the most well-established mechanisms of Aβ-induced toxicity. Therefore, we analyzed different mitochondrial markers: dynamin-related protein 1 (DRP1), optic atrophy-1 (OPA1), Peroxisome proliferator-activated receptor gamma coactivator 1- α (PGC1-α), and *phosphatase and tensin homolog* (PTEN). Our results showed that 5xFAD-treated mice presented a significant reduction in DRP1 protein expression, which takes part in the mitochondrial fission process, compared with 5xFAD control animals (P = 0.0277, Fig. 10A, C). Moreover, when we analysed the OPA1 protein expression required for the fusion of the mitochondria, we observed that 5xFAD control mice presented significantly lower levels of expression compared with 5xFAD UB-BJ-02 animals (P = 0.0386, Fig. 10A, B). In the same way, *PGC1-α* gene expression, the master transcription regulation that stimulates mitochondrial biogenesis, presented a tendency to be higher in 5xFAD-treated mice compared to the 5xFAD control group (P = 0.0811, Fig. 10D). Furthermore, PTEN gene expression, a crucial mediator of mitochondria-dependent apoptosis, significantly decreased in 5xFAD UB-BJ-02 compared to the 5xFAD control (P = 0.0282, Fig. 10E). These findings demonstrated that the sEH inhibition led to beneficial effects in mitochondrial dysfunction, increasing and modulating the biogenesis and fusion of the mitochondria.

### sEH inhibition by UB-BJ-02 brain effects correlate with microbiota changes

**Figure 11.**
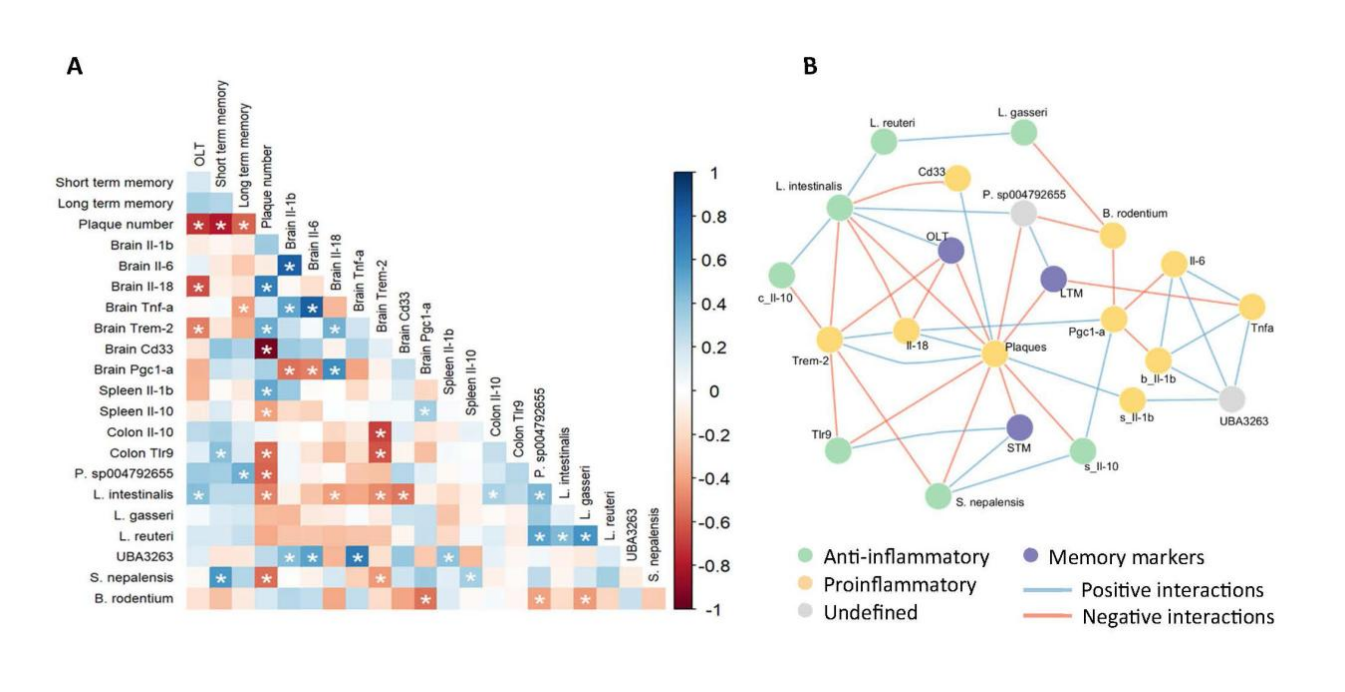
**Integrated correlation and network analysis of gut microbiota, immune markers, and memory performance**. (**A**) Heatmap illustrates the pairwise Spearman correlation coefficients between biological markers and cognitive functions. The heatmap includes object location (OLT), short-term (STM) and long-term memory (LTM), Aβ plaquesd, cytokine expressions such as *Il-1β, Il-6, Il-18, Tnfα*, and *Il-10* from spleen, and colon, microglia markers and relative abundance of gut microbiota species. Statistically significant correlations are indicated by asterisks (p < 0.05). (**B**) The network diagram illustrates significant correlation connections between variables from the heatmap, with nodes color-coded according to their biological role: cognitive function (STM, LTM, OLT) in purple, proinflammatory factors in yellow, anti-inflammatory factors in green, and factors with undefined properties in grey. Edge colors distinguish positive (blue) from negative (orange) correlation types, encapsulating the interplay among gut microbiota, immune mediators, and cognitive performance.

When 5xFAD mice were treated with UB-BJ-02, differential increases in specific microbial genera were observed, including *L. intestinalis*, *S. nepalensis*, and *Prevotella sp004792655*. Notably, improvement in cognitive function correlated positively with the prevalence of these genera while correlating negatively with the abundance of Aβ plaques in brain tissue (*L. intestinalis* correlated positively with OLT, P = 0.0023; *P. sp004792655* correlated positively with long-term memory (LTM), P = 0.007; *S. nepalensis* correlated positively with short-term memory (STM), P = 0.0012; Aβplaques correlated negatively with OLT, P = 0.010; STM, P = 0.006; and LTM, P = 0.027; Fig. 11A, B). In addition, significant associations were identified between cognitive performance and microbial communities, as well as various systemic and gut biomarkers (Colon Tlr9 correlated positively with STM, P = 0.041; and negatively with amyloid plaque, P = 0.028; spleen Il-10 correlated negatively with amyloid plaque, P = 0.046; spleen *Il-1β* correlated positively with them, P = 0.033; Fig. 11A, B). These findings suggest potential mechanisms by which UB-BJ-02 may exert its effects, including increased production of anti-inflammatory cytokines such as interleukin-10 (IL-10). Conversely, increases in pro-inflammatory markers and Aβ plaque formation were associated with the upregulation of other microbes, including *B. rodentium* and *UBA3263*, in the 5xFAD model (*UBA 3263* correlate with brain *Tnf-α*, P = 0.011; brain *Il-1β*, P = 0.045; brain *Il-6*, P= 0.037; spleen *Il-1β*, P = 0.047; *B. rodentium* correlates with *Pgc1-α*, P = 0.052 Fig. 11A, B).

### Dietary changes in CL4176 strains by bacterial genera may modulate the hallmarks of AD

**Figure 12.**
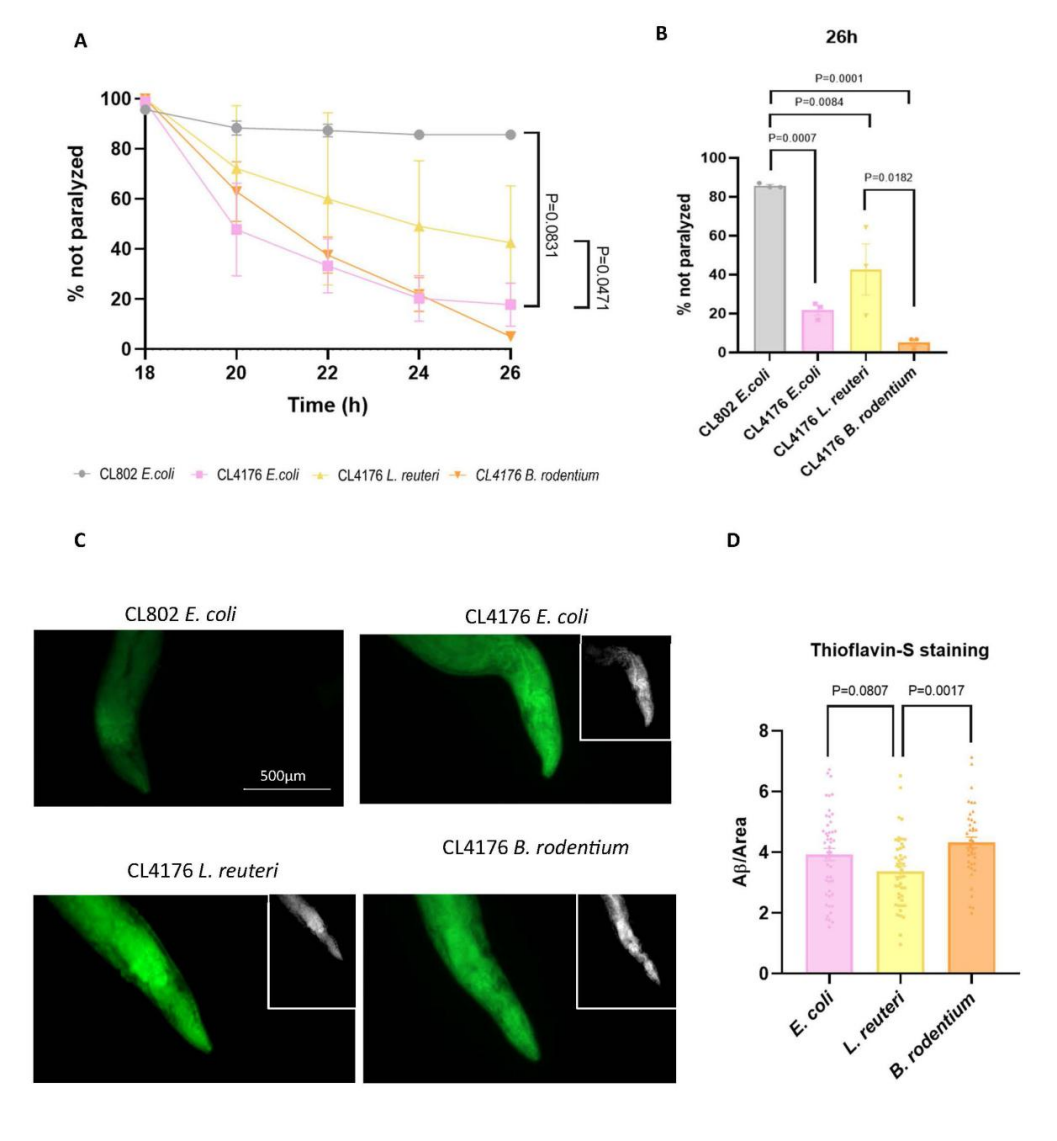
**Modulation of Aβ plaques in CL4176 strain**. (**A**) Paralysis assay graph and (B) % of worms are not paralyzed after 26 hours. (**C**) Representative images of Aβ plaques stained with Th-S and (D) quantitative analysis of Th-S staining. Results are expressed as a mean SEM. Groups were compared using the One-Way ANOVA test and post-hoc Tukey’s test (n=3/group).

A paralysis assay was performed with CL4176 *C. elegans* AD transgenic strain. The CL802 control strain was fed with *E. coli* and reached 85% survival, whereas CL4176 control worms also fed with *E. coli* achieved 17.5% survival 26 hours post temperature up-shift (P = 0.0007, Fig. 12A, B). In contrast, the worm survival rates were further increased when CL4176 were fed with *L. reuteri*, reaching 42,4% survival at 26 hours. Finally, CL4176 worms fed with *B. rodentium* presented a lower survival percentage at 26 hours compared to *L. reuteri*, with only 5% survival (P=0.0182, Fig. 12A, B). To support these results, Th-S staining evaluated Aβ deposits on the head regions of CL4176 worms. As expected, CL802 worms did not present Aβ aggregates, whereas CL4176 fed with *E. coli* exhibited an elevated number of Aβ plaques. Nevertheless, when worms fed with *L. reuteri* showed a decrease in the number of Aβ plaques compared with worms fed with *E. coli* (P = 0.0807, Fig. 12C, D). Conversely, *B. rodentium* significantly increased the number of Aβ plaques on CL4176 worms compared with *L. reuteri* (P = 0.0017, Fig. 12C, D), even exceeding the number of Aβ plaques found in worms fed with *E. coli*.

**Figure 13.**
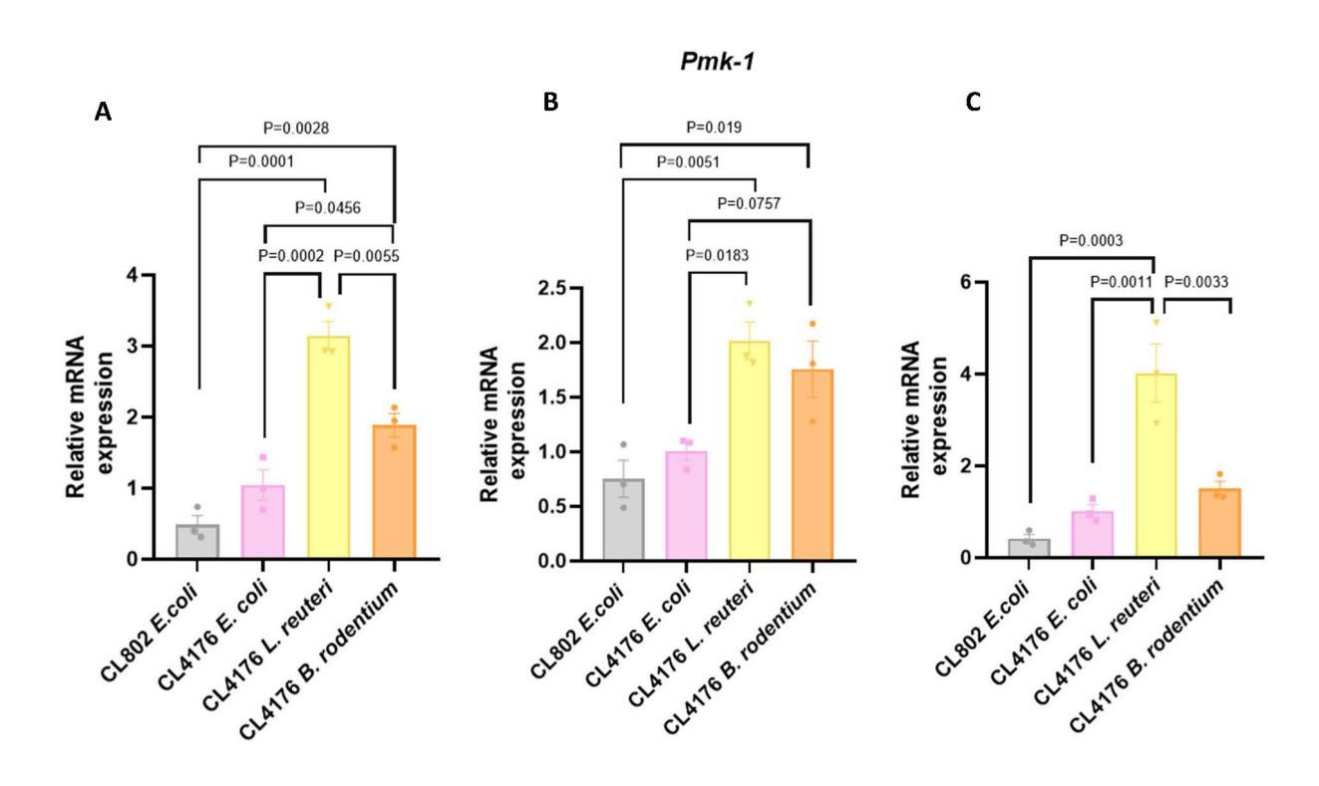
**Dietary changes in CL4176 strain by bacterial genera can modulate neuroinflammation**. Representative gene expression of (A) *Tol-1*, (B) *Pmk-1* and (C) *Skn-1*. Results are expressed as a mean SEM. Groups were compared using the One-Way ANOVA test and post-hoc Tukey’s test (n=3/group).

Moreover, we evaluated several genes involved in the p38-MAPK pathway, a signal transduction cascade in nematode immunity that plays a central role in the response against different pathogens [36-38]: toll-like receptor homolog (*Tol-1*), p38 mitogen-activated protein kinase-1 homolog (*Pmk-1*), and skinhead family member-1 (*Skn-1*). We observed that *L. reuteri* presented significantly higher gene expression of those pro-inflammatory markers compared with CL4176 *E. coli*-fed worms (P = 0.0002, P = 0.0183 and P = 0.0011, respectively, Fig. 13A-C). However, a reduction of gene expression was found when worms were fed with *B. rodentium* compared with *L. reuteri*, reaching significance in *Tol-1* and *Skn-1* (P = 0.0055 and P =0.0033, Fig. 13A, C). However, these gene levels were higher than the worms fed with *E. coli*, suggesting only partial activation of the p38-MAPK pathway with *B. rodentium* (P = 0.0456 and P=0.0757, Fig. 13A, B).

## DISCUSSION

Recently, it has been discovered that elevated inflammatory marker levels in patients with AD and several AD risk genes are associated with the innate immune system, suggesting that neuroinflammation has an important role in the etiopathogenesis of the disease [39, 40]. Besides, it has been reported that Aβ could be considered as a cytokine, unifying for the first time the neuroinflammation and amyloid pathology hypotheses [8]. Thus, targeting neuroinflammation could be an excellent strategy to stop AD progression. In this context and based on previous results from our group [17], the pharmacological inhibition of the sEH could be a therapeutic approach, preventing neuroinflammation processes and thereby stopping neurodegeneration. However, the effects of sEH inhibition in peripheral inflammation and, particularly, modulating the gut microbiota composition in AD models are less well described. Additionally, it has been reported that during the aging process as well as in neurodegenerative diseases, there are significant changes in the balance and diversity of the microbiota, a phenomenon called dysbiosis, which might affect blood-brain barrier (BBB) permeability, altering synaptic plasticity and promoting neuroinflammation and the formation of β-amyloid plaques [41]. In fact, recent studies have revealed a close connection between the gut microbiota and the neuroinflammatory processes that contribute to the pathogenesis of AD through the well-known gut-brain axis [42-46]. Therefore, understanding and modifying the microbiota composition might offer new therapeutic perspectives to treat the disease [47].

In this study, we provided compelling data after sEH inhibition evidencing the role of gut dysbiosis in the modulation of peripheral or systemic inflammation, which might reduce neuroinflammation in a mouse model of AD (5xFAD). To do that, we used a selective and potent sEH inhibitor, UB-BJ-02 [22], to confirm its beneficial effects and to further study the mechanisms driving neuroprotection in the AD model. In this way, we first evaluated the impact of the sEH inhibition on microbiota composition. Here, we demonstrated a reduced α-diversity in microbiota of 5xFAD mice; this imbalance in the gut microbiota promotes peripheral inflammatory activation. Indeed, this is consistent with observations in other mouse models [48, 49] and findings in AD patients [50]. To evaluate whether gut microbiota composition is modulated by sEH inhibition, we examined the gut microbiota fecal samples of 5xFAD after UB-BJ-02 treatment and, notably, we found effectively preserved microbiota diversity.

There is a known correlation between higher α-diversity in the microbiota and better cognitive performance [51], which we also observed in our study. However, neither genetic differences in the mouse strains nor treatment with the sEH inhibitor significantly altered the β-diversity index between groups. Untreated 5xFAD mice showed a reduction in the genera *Lactobacillus*, *Prevotella* and *Limosilactobacillus*, accompanied by a higher prevalence of the genus *UBA3263* of the family Muribaculaceae and the species *Bacteroides rodentium*. These changes in the microbiota were comparable to those observed in SAMP8 mice, another model of AD [52]. Alterations in the gut microbiota have been linked to changes in the host metabolome that affect brain function and contribute to neurodegenerative diseases [53]. Gut microbiota-derived metabolites may act as positive modulators of the gut-brain axis, strengthening immune cells and exerting protective effects against the progression of neurodegenerative diseases [54]. Conversely, disrupting the microbial balance may produce harmful metabolites associated with neurodegeneration. For example, dysregulation of Gram-negative bacteria leads to detrimental metabolites such as endotoxins and amyloids [55]. sEH inhibition after UB-BJ-02 treatment significantly prevented these alterations, suggesting that its protective effect involves changes in the microbiota profile.

In untreated 5xFAD mice, there was an increased abundance of the *UBA3263* genus, which was positively associated with increased *Il-1β* expression at both the brain and spleen levels. However, its concentration was unchanged in plasma. This bacterial genus also correlated positively with increased expression *of Tnf-α* and *Il-6* in the brain. These results agree with those previously reported by Künstner et al. [56], in which this genus correlated with shorter life expectancy and increased susceptibility to metabolic diseases. Furthermore, it was observed that the presence of this bacterium correlated with decreased expression of *Pgc1-α*, which plays a very relevant role in protection against oxidative stress and neuronal damage [57]. Likewise, members of the genus *Bacteroides* have been observed to contribute to the pathogenesis of AD by suppressing the phagocytic function of microglia, leading to impaired clearance of Aβ and accumulation of amyloid plaques. For instance, administration of *B. fragilis* resulted in an increased amyloid plaque burden in the cortex of APP/PS1 mice [58]. The effect of the sEH inhibitor, UB-BJ-02, in reducing these taxa could reduce the proinflammatory profile in 5xFAD mice.

Dysbiosis is also recognized to influence the inflammatory response [59]. In this regard, this study demonstrated a significant positive correlation between the abundance of *Lactobacillus intestinalis* and colonic *Il-10* expression, which correlated with improved cognitive performance. Furthermore, the expression of proinflammatory cytokines, such as *Il-18*, and microglial activation markers, including *Cd33* and *Trem-2*, are inversely related to *L. intestinalis* abundance. These results are consistent with previous research by Azm et al. [60], which demonstrated improved learning and memory in mice following *Lactobacillus* supplementation in a model of Aβ-induced cognitive decline. At the mechanistic level, the interaction between gut microbiota and the brain likely involves immune modulation and activation of neuronal and endocrine pathways by microbial metabolites [61]. In our 5xFAD model, a decrease in *Lactobacillus* abundance was linked to reduced *IL-10* expression in the colon, paralleled by lower expression of TLR9 in the colon mucosa. Indeed, research has demonstrated that *Lactobacillus* species can exert their anti-inflammatory effects by activating the TLR9 receptor [62]. Activation of TLR9 initiates a signaling cascade that promotes an anti-inflammatory profile, effectively contributing to the reduction of inflammation across multiple experimental colitis models [63]. It is of note that the administration of UB-BJ-02, then inhibiting sEH, prevented the decrease in *Lactobacillus* abundance and the associated decrease in TLR9 and IL-10 expression. This less inflammatory environment may improve the integrity of the intestinal barrier, thereby decreasing the likelihood of translocation of bacteria or bacterial products across the intestinal mucosa, which may lead to subacute or chronic inflammation [64].

Once the peripheral changes were evaluated, we focused on the modifications produced by the sEH inhibition in the CNS. A typical feature of AD is cognitive decline, first recognized as memory impairment [65]. As expected, we showed that 5xFAD-treated mice presented better short- and long-term memory and spatial memory in comparison with the 5xFAD control. Based on these findings, we can affirm that sEH inhibition by UB-BJ-02 reduced the cognitive impairment presented in 5xFAD mice. In line with our results, previous studies and reports have described the positive effects generated by sEH inhibition in different neurodegeneration mice models [17, 66-69].

Afterward, it was interesting to evaluate other parameters that could explain the cognition improvement in the UB-BJ-02 treated group, then related with sEH inhibition. In this way, we analyzed a well-established pathological hallmark of AD, the Aβ plaques, by Th-S staining. Importantly, the number of plaques in 5xFAD mice significantly decreased after UB-BJ-02 treatment, from 350 to 250 plaques, representing a 29% reduction. In line with our results, other studies of our group showed the high among of Aβ depositions that present 5xFAD mice [19] and its reduction after the inhibition of the sEH with other compounds, such as TPPU, UB-EV-52 or UB-SCG-51 [17, 70]. Additionally, a recent study linked to this data showed that treating 5xFAD with antibiotics or probiotics for 14 weeks led to gut microbiome changes associated with lower Aβ plaque levels and improved cognitive function [71], indicating the gut microbiome's role in 5xFAD's neurodegeneration. Considering Aβ accumulations as proinflammatory cytokines, we also analyzed changes in other neuroinflammatory markers after sEH inhibition. Neuroinflammation, driven by activated microglia and astrocytes and involving interleukins, plays a key role in AD pathogenesis, contributing to synaptic dysfunction, neuronal loss, and death [72, 73]. As we expected, our data showed that the treatment significantly reduced the astrogliosis and microgliosis through GFAP and IBA-1 reduction as well as the microglia and astrocyte release of interleukins, such as *Il-1β, Il-6*, or *ll-18* and the transcription factor *Tnf-α*, which is a pivotal molecule in inflammation. It also has been shown to play an important role in AD pathogenesis, exacerbating both Aβ and Tau hallmarks *in vivo*. Interestingly, anti-inflammatory strategies, such as TNF-α inhibitors, have ameliorated cognitive function in rodent models of AD, attenuating the characteristic brain pathology [74, 75].

Additionally, our results show decreased *Trem2* and increased *CD33* expression after the UB-BJ-02 treatment in the 5xFAD mice. *Trem2* might be protective in later stages by reducing amyloid accumulation and neuroinflammation [76, 77]. In contrast, *CD33* inactivation has been proposed to mitigate amyloid pathology by modulating microglial activity [78]. The changes in *Trem2* and *CD33* expression could reflect an adaptive immune response in the advanced stages of the disease. These findings suggest that the modulation of *Trem2* and *CD33* may be part of the neuroprotective mechanisms driven by sEH inhibition, restoring the neuroinflammatory process and reducing the amyloid burden.

To further study the beneficial effects of sEH inhibitor UB-BJ-02 in the brain, we also studied the consequences of its inhibition in mitochondrial dysfunction. Excessive mitochondrial fission can lead to brain dysfunction, and it has been associated with the Aβ accumulation in the brains of patients and, therefore, with the pathophysiology of several neurodegenerative diseases [79]. In this way, we analyzed OPA1 and DRP1, respectively [80, 81]. Of note, AD is characterized by reduced OPA1 [82] and increased DRP1 protein levels [83]. This imbalance results in premature age-related loss of spines in hippocampal neurons, contributing to synaptic dysfunction, neuronal damage, and cognitive decline. We observed that OPA1 increased significantly after the treatment, whereas DRP1 decreased, suggesting that mitochondrial fusion increased, whereas fission decreased by sEH inhibition.

Additionally, treatment benefits on mitochondria are indicated by an increase in *Pgc1-α* and a decrease in *Pten*. *Pgc-1α* upregulation has been shown to be neuroprotective against oxidative stress and cell damage by improving mitochondrial function, neuroinflammation, protein clearance, and neuronal maintenance [84]. Conversely, *Pten* shows an increase of its expression as AD progresses, correlating with a significant reduction in synaptic density [85]. Additionally, elevated levels of phosphorylated Tau in AD trigger the early activation of *Pten*, preceding the initiation of the apoptotic pathway [86]. To reinforce the remarkable results on the modulation of the gut-brain after sEH inhibition and to further demonstrate the narrow correlation among the beneficial effects shown by sEH inhibitor treatment in 5XFAD, we used the *C. elegans* CL4176 AD strain to investigate the impact of microbiota alteration on AD hallmarks. We found that nematodes fed with *L. reuteri* showed significant behavioral improvements and a lower percentage of paralysis compared to those fed with *E. coli* or *B. rodentium*, with B. *rodentium*-fed worms displaying the highest paralysis rate. Additionally, *L. reuteri* was observed to reduce Aβ plaque presence, a key factor in AD pathology, more effectively than *B. rodentium* or E. coli. Moreover, *L. reuteri* feeding led to a notable activation of the p38 MAPK pathway, known for its role in neuroprotection and immune function [87], unlike *B. rodentium*. These findings suggest that feeding nematodes with beneficial bacterial gender can lead to behavioral changes, reduced Aβ plaques, and enhanced immune system activation, whereas *B. rodentium* drives deleterious effects. Those results support previous research conducted on 5xFAD mice and highlight the potential neuroprotective effects of *L. reuteri.*

**Figure 14.**
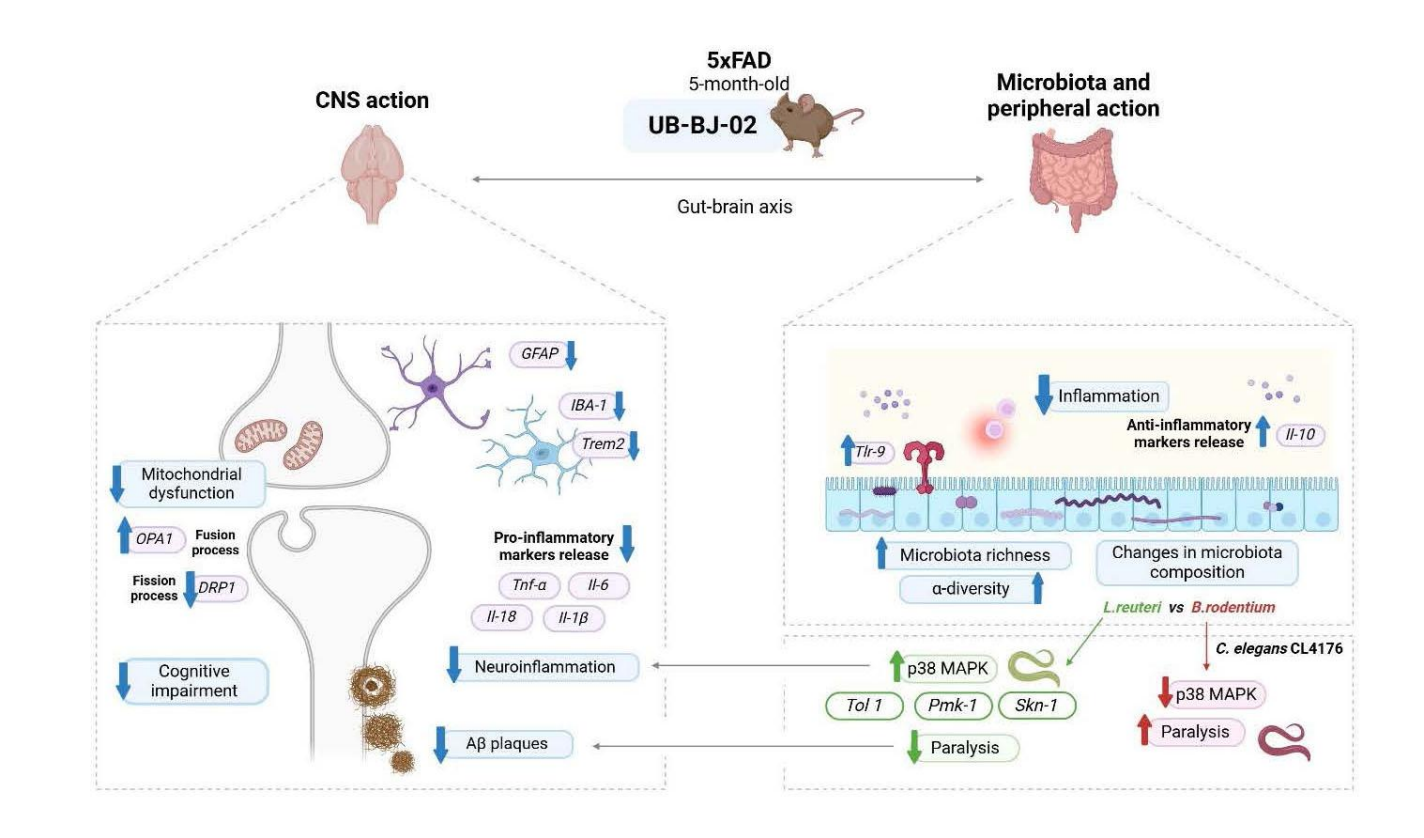
Illustrative scheme of the UB-BJ-02 effects in 5xFAD mice.

## Conclusions

In conclusion, we added knowledge to the described neuroprotective effects after sEH inhibition UB-BJ-02. This compound triggers a string of neuroprotective actions in the 5xFAD mouse model, modulating gut microbiota and peripheral inflammation as well as improving mitochondrial dynamics and reducing neuroinflammation. Because of the positive correlation between peripheral improvements and central outcomes, such as enhanced cognition and reduced neuroinflammation [88, 89], they can be highlighted after sEH inhibition in 5xFAD. Those findings emphasized the microbiota and peripheral inflammation's significant role in neurodegenerative diseases and, particularly, their influence on AD hallmarks [90-92], suggesting that the gut microbiome significantly impacts in its pathology development and that sEH inhibition has a beneficial effect on AD mice model implicated gut-brain axis modulation (Fig. 14). In addition, the inclusion of metabolomic analyses in future research may further our understanding of the mechanisms by which sEH enzyme inhibition exerts its neuroprotective effects, as sEH inhibition may affect levels of EETs and their metabolites, which have anti-inflammatory and neuroprotective properties.

## Supplementary Materials

The Supplementary data can be found online at: www.aginganddisease.org/EN/10.14336/AD.2025.0201.

## Data Availability

The datasets generated during and/or analysed during the current study are not publicly available but are available from the corresponding author on reasonable request. The raw sequencing data from the microbiota analysis conducted in this study have been deposited in Zenodo and are publicly available at DOI: 10.5281/zenodo.15023907.
