# Supplementary material for: Soluble Epoxide Hydrolase Inhibition Improves Alzheimer’s Disease Hallmarks: Correlation with Peripheral Inflammation and Gut Microbiota Modulation
Source: Aging Dis. 2025 Mar 23;17(2):1131–54. doi: 10.14336/AD.2025.0201 (PMC12834399; doi:10.14336/AD.2025.0201)
Supplement: Supplementary file 1 — The Supplementary data can be found online at: www.aginganddisease.org/EN/10.14336/AD.2025.0201. [file AD-17-2-1131-s.pdf]

SUPPLEMENTARY DATA

Soluble Epoxide Hydrolase Inhibition Improves Alzheimer's Disease Hallmarks: Correlation with Peripheral Inflammation and Gut Microbiota Modulation

Júlia Jarne-Ferrer, Christian Griñán-Ferré, Beatrice Jora, Sandra Codony, Lluïsa Miró, Cristina Rosell-Cardona, David Miñana-Galbis, Anna Pérez-Bosque, Santiago Vazquez, Mercè Pallàs

© 2025. Jarne-Ferrer J et al. Published online at http://www.aginganddisease.org/EN/10.14336/AD.2025.0201

SUPPLEMENTARY DATA

Supplementary Table 1. Primer sequences of real-time qPCR (SYBR Green primers).

Primer	Forward (5'-3')	Reverse (3'-5')	Size (bp)
Il-1β	GGTCAAAGGTTTGGAAGCAG	TGTGAAATGCCACCTTTTGA	94
Il-10	GGCGCTGTCATCGATTTCTCCCC	TGGCCTTGTAGACACCTTGGTCTT	102
Il-6	TGTGAAATGCCACCTTTTGA	GGTCAAAGGTTTGGAAGCAG	109
Myd88	TGCCAGCGAGCTAATTGAGAA	TGCCAGCGAGCTAATTGAGAA	130
Tlr2	GTTTCTGAGTGTAGGGGCTTCA	CATCCTCTGAGATTTGACGCTTTG	122
Tlr4	GCTTGAATCCCTGCATAGAGGTAG	GAAGAAGGAATGTCATCAGGGACT	91
Tlr9	GAGAGACCCTGGTGTGGAAC	CCTTCGACGGAGAACCATGT	98
Tnf-α	CCACCACGCTCTTCTGTCTAC	AGGGTCTGGGCCATAGAACT	103
Hprt1	TGGATACAGGCCAGACTTTGTT	TGGATACAGGCCAGACTTTGTT	163
Trem2	CCTGAAGAAGCGGAATGGG	CTTGATTCCTGGAGGTGCT	269
CD33	GTCAACCACCCAACCTTCCT	ATGGACACATAGGCCCTCCT	175
Pgc1-α	ATTCGGCACGAGGTTGCC	TGACGCCAGTCAAGCTTTTT	78
Pten	CCGGTGTTAAGCCTCCCGTC	TCTGCAGGATGGAAATGGCTC	277
**β-actin**	CAACGAGCGGTTCCGAT	GCCACAGGTTCCATACCCA	66
Tol-1	CCCAGTTTGAGATCACATGACA	TTCGACGCAATAGGTGT	23
Skn-1	CCAACATCCAACTACGCCT	GTCTGCTGTTGACGTCCTGA	29
Pmk-1	GGTCATCGTTGAGTCGCTGA	TGCCCGTCGTACATATCGTG	20
Act-1	ATCACCGCTCTTGCCCCATC	GGCCGGACTCGTCGTATTCTTG	151

Supplementary Table 2. Antibodies used in Western blot studies.

Antibody	Host	Source/Catalog	Dilution
**Opa1**	Mouse	Santa Cruz/sc-3932396	1:500
**Drp1**	Mouse	BD Bioscence/611738	1:1000
**Actin**	Mouse	Sigma Aldrich/A2228	1:2500
**Goat-anti-mouse HRP conjugated**		Biorad/170-5047	1:2000

© 2025. Jarne-Ferrer J et al. Published online at http://www.aginganddisease.org/EN/10.14336/AD.2025.0201
